# Single-cell Deciphering of the Progression Trajectories of the Tumor Ecosystem in Laryngeal Squamous Cell Carcinoma

**DOI:** 10.7150/ijbs.129291

**Published:** 2026-03-28

**Authors:** Zhimou Cai, Yun Li, Jinhong Zhang, Shiyun Luo, Zhiwei Qiang, Zhaoyue Lu, Lin Chen, Wenbin Lei

**Affiliations:** Department of Otolaryngology, The First Affiliated Hospital of Sun Yat-sen University, Guangzhou, Guangdong, P.R. China

**Keywords:** laryngeal squamous cell carcinoma, single-cell RNA sequencing, tumor microenvironment, POSTN^+^ fibroblast, macrophage migration inhibitory factor

## Abstract

Laryngeal squamous cell carcinoma (LSCC) is typically diagnosed at advanced stages, highlighting the critical need for early intervention. By integrating single-cell and bulk RNA-seq data from LSCC, vocal cord leukoplakia (VCL), and LSCC precursors, we characterized dynamic remodeling of the tumor microenvironment during LSCC pathogenesis. We identified transcriptional program gene modules that reflect malignant epithelial cells (maEpCs). The infiltration of POSTN^+^ fibroblasts progressively increases from normal tissue to VCL and further to LSCC, accompanied by enhanced intercellular communication. These fibroblasts interact with maEpCs and endothelial cells via ligands such as MIF, promoting epithelial-mesenchymal transition, cancer stemness, and angiogenesis. Blocking MIF reversed cancer-associated fibroblast-driven invasion and angiogenesis. Here, we further revealed that an immunosuppressive microenvironment arises as early as the precancerous stage, with VCL exhibiting CD8^+^ T cell exhaustion and abundant LAMP3^+^ dendritic cells that correlate positively with Tregs and exhausted CD8^+^ T cells, promoting early immune escape. Additionally, LSCC was uniquely enriched for a pro-tumor SPP1^+^ macrophage subset with low phagocytic activity and high angiogenic potential, linked to poor prognosis. Our findings uncover key mechanisms driving LSCC malignant progression, offer insights for early diagnosis and prognosis assessment, and highlight MIF as a promising therapeutic target.

## Introduction

Laryngeal squamous cell carcinoma (LSCC) originates from the laryngeal squamous epithelium and ranks as the second most prevalent tumor in the head and neck region 1. Owing to inconspicuous early symptoms, approximately 60% of the patients are diagnosed at advanced stages, missing the optimal treatment window. The efficacy of conventional therapies has remained unsatisfactory for decades, with stagnant survival rates and poor prognoses in advanced diseases 2. Furthermore, aggressive treatments for advanced LSCC often cause serious side effects, such as difficulty swallowing, airway changes, and loss of voice, leading to high disability, significant psychological problems, and greatly reduced quality of life 3. Therefore, the early identification of high-risk precancerous lesions, prompt detection of laryngeal cancer, and implementation of effective interventions are essential for enhancing clinical outcomes.

Approximately 90% of patients with LSCC develop from precancerous lesions, among which vocal cord leukoplakia (VCL) is the most frequently observed 4. Such established clinical progression underscores the vital window of opportunity for early intervention. VCL presents as white plaques adherent to the vocal cord mucosa, with pathological manifestations ranging from inflammatory or epithelial hyperplasia to varying grades of dysplasia or even carcinoma *in situ* 5. The histological heterogeneity of lesions plays a crucial role in guiding clinical management decisions. Non-atypical lesions may be managed conservatively. However, atypical lesions require surgical excision due to their higher recurrence rates and the increased risk of malignant transformation with greater degrees of atypia. In certain cases, this may even warrant the consideration of radiotherapy as part of the treatment plan 4,6,7. Despite its clinical significance, mechanistic studies on the carcinogenic transformation of VCL remain limited, and the precise molecular mechanisms underlying its progression to LSCC remain unelucidated 8. The existing knowledge gap poses a challenge in clinical practice, impeding our capability to accurately predict malignant potential and effectively stratify patient risks. Consequently, establishing more precise histopathological diagnostic criteria for VCL and conducting in-depth molecular biology investigations are crucial for the timely assessment of cancer risk, evaluation of prognosis, and guiding optimal therapeutic decisions.

Single-cell RNA sequencing (scRNA-seq) enables high-resolution analysis of cellular heterogeneity, identification of rare cell subpopulations, and discovery of molecular regulatory networks within complex tissues 9. With the widespread application of this technology, researchers have progressively begun to characterize the cellular composition and functional diversity of LSCC ecosystems at single-cell resolution. Wei *et al.* 10 first described the cellular atlas of LSCC using scRNA-seq. Yan *et al.* 11 and Shen *et al.* 12 constructed single-cell atlases of LSCC and its lymph node metastases, further elucidating the cellular architecture, functional states, and intercellular communication networks within the tumor immune microenvironment (TIME) and delineating cancer stem cell characteristics in LSCC. Notably, scRNA-seq enabled us to overcome the limitations of conventional bulk RNA sequencing and to precisely capture dynamic transcriptomic changes across different stages of LSCC initiation and progression. Recently, Zhou *et al.* 13 produced the first single-cell atlases of vocal cord polyps (VCP) and VCL, which laid the groundwork for comparing cellular heterogeneity across benign, premalignant, and malignant laryngeal lesions, thereby providing a new framework for understanding LSCC pathogenesis. Although these studies provided valuable preliminary insights into the cellular heterogeneity of LSCC and its precancerous lesions, several key scientific questions regarding its initiation and progression remain unresolved. First, the dynamic evolution of the tumor microenvironment (TME) throughout the multistep progression from normal tissue to precancerous lesions and ultimately to invasive carcinoma remains inadequately understood. Investigating whether this progression is influenced by specific critical cellular subsets that facilitate malignant transformation is essential. Second, the conserved or specific biological programs, as well as the similarities and differences in oncogenic pathways between VCL and LSCC tumor cells, remain unknown. Third, as tumor cells, fibroblasts, endothelial cells, myeloid cells, and T cells are the most significant cell types within the LSCC TME, the mechanisms underlying their interactions and the mechanisms by which their collective signaling drives disease progression remain unknown.

A comprehensive understanding of this multistep carcinogenesis process is critical for understanding the mechanisms of tumor evolution and identifying targets for early intervention 14. To address these questions, we conducted an in-depth reanalysis of the scRNA-seq dataset published by Zhou *et al.* 13 to systematically delineate the dynamic remodeling of the TME during LSCC progression and elucidate the key mechanisms driving LSCC development. By evaluating the infiltration abundance and biological functions of malignant, immune, and stromal cells across disease stages, we reconstructed the developmental trajectory of LSCC. Furthermore, focusing on cell-cell communication, we compared the critical differences in communication between precancerous lesions and tumors and identified core intercellular signaling pathways that promote malignant progression, underpinning the malignant transition from precancerous lesions to invasive LSCC. Finally, by integrating multiple bulk RNA-seq datasets and performing functional experiments, we validated the findings in multiple dimensions, thereby providing a more comprehensive interpretation of the mechanisms underlying tumor initiation and progression within the LSCC ecosystem. In this study, we not only provide novel perspectives for identifying diagnostic biomarkers and therapeutic targets in LSCC but also advance early intervention and precision treatment strategies for laryngeal precancerous lesions, thereby benefiting a broader patient population.

## Materials and Methods

### Data acquisition

The scRNA-seq data were downloaded from the Gene Expression Omnibus (GEO, https://www.ncbi.nlm.nih.gov/geo/, GSE157220), which contained data from 10 VCP, 10 VCL, and 10 paired LSCC precursor (LSCCP) and LSCC samples (totaling 30 patients), generated using the 10× Genomics Chromium system 13. Relevant clinical information and bulk RNA-seq data regarding patients with LSCC were obtained from the Cancer Genome Atlas database (TCGA, https://portal.gdc.cancer.gov/, TCGA-HNSC) and the GEO database (GSE25727 15, GSE27020 16, and GSE65858 17). The GSE25727 and GSE27020 datasets were selected as they specifically contain LSCC data. For a more accurate analysis, only the LSCC portion was extracted from the TCGA-HNSC and GSE65858 datasets and named TCGA_LSCC and GSE65858_LSCC, respectively.

### Quality control and batch effect correction of scRNA-seq data

All scRNA profile analyses were conducted according to the standard workflow provided by the Seurat package (v5.1.0) in R (v4.4.1) 18. Cells with a mitochondrial gene percentage of > 15%, a hemoglobin gene percentage of > 1%, or < 500 detected genes were removed due to low quality. To eliminate potential doublets, cells with > 5,000 detected genes were removed. Furthermore, the DoubletFinder package (v2.0.4) was used to detect and remove potential doublets 19. The expected doublet rate for each sample was estimated individually using a Poisson distribution with the calcDBRate function, accounting for sample-specific cell numbers. This estimated doublet rate was explicitly provided to DoubletFinder for each sample, while other parameters were kept at their default settings. Cells predicted as doublets were excluded from downstream analyses.

Following quality control, the UMI count matrix was normalized using the NormalizeData function, and highly variable genes were identified using FindVariableGenes. The data were then scaled using ScaleData, and principal component analysis (PCA) was performed using RunPCA to achieve linear dimensionality reduction. To integrate cells across multiple samples for unsupervised clustering, batch effects were corrected utilizing the Harmony algorithm (v1.2.1) in the R package 20.

### Dimensionality reduction and clustering

Using thirty principal components at a resolution of 1.0, cells were clustered by employing the Seurat package's FindNeighbors and FindClusters functions. The cells were visualized by UMAP embedding. Classification of cells into known types was conducted with reference to conventional marker genes described in previous studies 21.

### Copy number variant (CNV) analysis for epithelial cells

The InferCNV R package (v1.20.0) 22, with default parameters, was utilized to identify large-scale chromosomal CNVs in epithelial cells and to distinguish malignant from non-malignant cells. Immune and stromal cells served as reference cells. K-means clustering based on CNV scores was performed on epithelial cells, along with reference cells from the LSCC and VCL groups. Epithelial cells clustered with reference cells were identified as non-malignant, whereas those exhibiting elevated CNV scores in separate clusters were designated as malignant epithelial cells (maEpCs).

### High-dimensional weighted gene co-expression network analysis (hdWGCNA)

The hdWGCNA R package (v0.4.08) 23 was used to elucidate the regulatory mechanisms underlying tumorigenesis and progression. maEpCs were identified based on CNV scores and extracted from the scRNA-seq data. By constructing gene expression correlation matrices, generating weighted gene co-expression networks, and identifying co-expression modules, gene modules associated with individual cell classifications and biological activities were explored. Using module-trait relationship analysis, associations between module eigengenes and maEpCs from either the LSCC or VCL groups were calculated, thereby identifying key modules that correlated significantly with disease status. Hub genes within these modules were identified based on intramodular connectivity.

### Pathway enrichment analysis

Differentially expressed genes (DEGs) in each cell subpopulation were identified using the FindMarkers function in the Seurat package, with the Wilcoxon rank-sum test. GSEA was performed using the R package fgsea (v1.30.0) 24 with genes ranked according to their average log2 fold change (log2FC) values to identify the latent enriched pathways in each cell subpopulation based on the KEGG and GOBP. For each gene set, the Normalized Enrichment Score (NES) was calculated, and the enrichment significance was determined using a permutation test with 10,000 iterations.

To estimate pathway activity in the cell groups, GSVA analysis was conducted using the GSVA R package (v1.52.3) 25. Hallmark gene sets were obtained from the Molecular Signature Database (http://www.gsea-msigdb.org). Differentially active pathways were identified using statistical analysis with the limma package in R.

### Signature score calculation

The signature scores were calculated using Seurat's “AddModuleScore” method, which averages scaled and centered gene expression values across predefined gene sets. The signature gene list included cytotoxicity and exhaustion scores for CD8^+^ T cells, M1 and M2 polarization scores for macrophages, and differentiation, activation, antigen presentation, migration, and tolerance scores for DCs. The genes involved are listed in the Supporting Information (Tables S1-3).

Cell type distributions in bulk-seq data were characterized by performing ssGSEA with the GSVA R package 25. This method calculated enrichment scores reflecting the proportion of cells of interest, based on predefined cell-type signatures derived from single-cell RNA sequencing.

### Correlation and survival analysis

Pearson correlation coefficients and corresponding p-values between the two target cell subpopulations were calculated using the cor.test function from the stats R package (v4.4.1). The surv_cutpoint function in the survminer R package (v0.4.9) was employed to stratify samples into high and low signature score groups by identifying the optimal cutoff. Prognostic evaluation was conducted through Kaplan-Meier survival analysis, with statistical significance assessed by the log-rank test.

### Cell developmental trajectory

The cell lineage trajectory of CD8^+^ T cells was inferred using the Monocle R package (v2.30.1) 26. A CellDataSet object was created from raw UMI counts using the negbinomial-size () expression family. Genes used for pseudotime ordering were selected based on cluster-specific DEGs identified using Seurat's FindAllMarkers function (log₂FC ≥ 0.1, adjusted p-value < 0.01). The cells were ordered along the trajectory using the DDRTree reduction method. Genes significantly associated with the pseudo-time progression were identified using the differential GeneTest function.

### Cell‒cell communication analysis

The signaling interactions between tumor cells and other cell populations were analyzed using the CellChat R package (v2.1.2) 27. The cell subpopulations were defined and grouped to create CellChat objects. Preprocessing steps were performed with default parameters, and the CellChatDB human ligand-receptor interaction database was used for analysis. The inference of networks for ligand-receptor pairs and signaling pathways was performed using the computeCommunProb and computeCommunProbPathway functions.

To achieve a more comprehensive characterization of intercellular communication, NicheNet analysis was next performed using the NicheNet R package (v2.2.0) 28 to link upstream ligands to downstream target gene regulation in receiver cells, thereby providing complementary functional insights into signaling consequences. And the differential NicheNet pipeline designed for condition-specific comparisons was applied to infer and compare differences in ligand-receptor interactions between cell subsets across different stages. Four niches, corresponding to four groups (LSCCP, VCP, VCL, and LSCC), were defined, each consisting of one sender cell type and one receiver cell type within the same group. The precomputed human ligand-receptor network and ligand-target matrix provided by NicheNet were used, retaining only bona fide interactions for subsequent analyses.

Differential expression analysis of senders and receivers was performed using calculate_niche_de, with an expression percentage threshold of 10%. A log2 fold-change cutoff of 0.25 was applied to define DEGs in receiver cells for ligand activity analysis. Ligand-receptor pairs were prioritized using a customized scoring scheme that integrated ligand and receptor expression, ligand-receptor specificity, and target gene activity. The top-ranked ligand-receptor pairs for each niche were selected for further visualization. To depict ligand expression, receptor regulation, and target gene activity, figures were produced using the make_ligand_receptor_lfc_plot and make_ligand_activity_target_exprs_plot functions.

### Immunofluorescence (IF) staining

Tissue sections were deparaffinized in xylene and rehydrated through a series of graded ethanol solutions. Antigen retrieval was performed by heat-induced treatment in either citrate or Tris-EDTA buffer (pH 6.0). After allowing the sections to cool to 25 °C, they were blocked with 5% goat serum for 1 hour. The sections were incubated overnight at 4 °C with the respective primary antibodies, including anti-collagen type I (COL1A1) (#TA422261M, OriGene, USA), anti-osteopontin (SPP1) (#ab9377, Abcam, USA), anti-wide spectrum cytokeratin (Pan-CK) (#TA385310M, OriGene), anti-periostin (POSTN) (#TA804575M, OriGene), and anti-CD68 (#66231-2-lg, Proteintech, China), followed by washing in phosphate-buffered saline (PBS). They were incubated with AlexaFluor-555 labeled goat anti-rabbit antibodies (#A- 21207, Invitrogen, USA) or AlexaFluor-488 labeled goat anti-mouse antibodies (#A-11001, Invitrogen) for 1 h, washed again in PBS, and counterstained with 4',6-diamidino-2-phenylindole (DAPI) to visualize the nuclei. Sections were imaged using a fluorescence or confocal microscope (Olympus/FV3000, Japan).

### Fibroblast culture and preparation of conditioned media (CM)

Fibroblast culture and CM generation were performed according to the protocols established in our previous studies 29. Briefly, primary fibroblasts were isolated from minced human LSCC tumor tissues via trypsin digestion, followed by centrifugation and resuspension in complete medium. The cells were cultured in a cell culture incubator (37 °C, 5% CO₂, and 95% humidity) and passaged at approximately 80% confluence. Fibroblasts in the logarithmic growth phase between passages 3 and 4 were used for subsequent experiments.

To prepare CM, fibroblasts were seeded at a density of 3 × 10⁴ cells per well in six-well plates in Roswell Park Memorial Institute (RPMI)-1640 medium supplemented with 10% fetal bovine serum (FBS). After 24 h, the medium was replaced with a serum-free RPMI-1640 medium and incubated for another 48 h. Supernatants were collected, centrifuged at 1,000 × *g* for 5 min at 4 °C, and designated as CAF^CM^ (from cancer-associated fibroblasts) or NF^CM^ (from normal fibroblasts).

When preparing CAF^CM^ containing Imalumab or IgG, a 1 mg.mL^-1^ stock solution of Imalumab was first diluted 1:100 in CAF^CM^ to achieve a final concentration of 10 μg.mL^-1^. Similarly, a working solution of the same concentration of IgG was prepared using CAF^CM^ as an isotype control. The CAF^CM^ + Imalumab or CAF^CM^ + IgG mixtures were preincubated at 37 °C for 30 min to allow the binding of Imalumab to macrophage migration inhibitory factor (MIF) present in the CAF^CM^.

### RNA extraction and quantitative real-time PCR (qPCR)

Total RNA was extracted from cells using TRIzol reagent and an RNA extraction kit (#AG21017, Accurate Biology, China). The cells included SNU899 and SNU1076 cells cultured for 48 hours in RPMI 1640 complete medium with or without recombinant human MIF (rMIF, #300-69-1MG, PeproTech, USA; 100 ng/ml), as well as treated macrophages. Reverse transcription was subsequently performed using a cDNA synthesis kit (#11119ES60, Yeasen, China). The resulting cDNA was subjected to qPCR using SYBR Premix Ex Taq II (#11202ES08, Yeasen). GAPDH was used as the endogenous reference gene, and relative expression levels of target genes were calculated using the 2^-ΔΔCt^ method. The primer sequences used in this study are listed in Table S4.

### Wound-healing and transwell invasion assays

For the wound-healing migration assay, SNU899 and SNU1076 cells were cultured for 24 h in six-well plates (#3335, Corning, NY, USA) in RPMI 1640 medium containing 10% FBS, and the cells were cultured to 95%-100% confluence. A uniform scratch was created in each well using a pipette tip. Subsequently, cells were rinsed twice with PBS and cultured for 24 h in serum-free RPMI 1640 medium supplemented with 25% NF^CM^, 25% CAF^CM^, 25% CAF^CM^ + Imalumab, 25% CAF^CM^ + IgG, rMIF (100 ng/ml)**,** or an equal volume of serum-free medium (vehicle control). Images documenting wound areas were taken immediately after scratching (0 h) and at 24 h. Migration distances were quantified using the ImageJ software (National Institutes of Health, Bethesda, MD, USA).

For the transwell invasion assay, SNU899 or SNU1076 cells were starved in serum-free medium for 24 h, following which cells (1 × 10⁵) in 200 μL serum-free RPMI-1640 medium containing 25% NF^CM^, 25% CAF^CM^, 25% CAF^CM^ + Imalumab, 25% CAF^CM^ + IgG, rMIF (100 ng/ml)**,** or an equal volume of serum-free medium (vehicle control) were plated into the upper chamber of 24-well transwell inserts (#3422, Corning, USA) pre-coated with Matrigel (#356234, BD Biosciences, USA). A medium supplemented with 20% FBS was placed in the lower chambers and incubated for 48 h to stimulate chemotactic migration. Cells that passed through the 8-μm pores were fixed with methanol and subsequently stained with 0.1% crystal violet. The stained cells were visualized using light microscopy, and migrated cells were quantified by counting in five randomly chosen fields per membrane.

### Protein extraction and western blotting

SNU899 and SNU1076 cells were cultured in RPMI-1640 medium containing 10% FBS for 24 h. Upon reaching approximately 60% confluency, the cells were gently rinsed twice with PBS and then incubated in serum-free RPMI-1640 medium containing 25% NF^CM^, CAF^CM^, CAF^CM^ + Imalumab, or CAF^CM^ + IgG. After 48 h, the cells were harvested and lysed in radioimmunoprecipitation assay buffer for protein extraction.

Protein concentration was determined using a bicinchoninic acid protein assay kit (#B6169, Uelandy, China). Equal amounts of proteins were separated using sodium dodecyl sulfate-polyacrylamide gel electrophoresis on FastPAGE precast gels (#TSP024, TsingKe, China) and transferred onto polyvinylidene fluoride membranes (#IPVH00010, Merck Millipore). The membranes were blocked with 5% non-fat milk and incubated overnight at 4 °C with primary antibodies. After washing, the membranes were incubated with horseradish peroxidase-conjugated secondary antibodies for 1 h at room temperature. The protein bands were visualized using an ECL detection system (#BL520B, Biosharp, China) and a MinChemi 580 imaging system (Saizhi, China). The antibodies used are listed in Table S5.

### Xenograft tumor model and treatment

Exponentially growing SNU1076 cells (1 × 10⁷ cells/mL) were subcutaneously injected into the right flank of BALB/c nude mice to establish a laryngeal squamous cell carcinoma xenograft model. Mice were randomly assigned to three groups (n = 4 per group). Two groups received intraperitoneal injections of either IgG (10 mg/kg) or imalumab (10 mg/kg) every 3 d.

Tumor growth was monitored every 3 d by measuring tumor length and width. Tumor volume was calculated using the formula V_T_ = (L × W²) / 2. On day 27, all mice were euthanized, and tumors were excised, measured, and weighed for further analysis. All animal experiments were approved by the Institutional Animal Care and Use Committee of Sun Yat-sen University (approval no. SYSU-IACUC-2026-000575).

### Generation of SPP1⁺ tumor-associated macrophage (TAM)-like macrophages and functional assays

THP-1 cells were maintained in RPMI-1640 complete medium. To generate adherent macrophages, THP-1 cells were differentiated with PMA (#HY-18739, MCE, USA) and allowed to recover, yielding unpolarized M0 macrophages. SPP1⁺ TAM-like macrophages were then induced using an indirect transwell co-culture system, in which M0 macrophages were non-contact co-cultured with SNU1076 or SNU899 cells. After co-culture, inserts were removed, and macrophages were transfected with SPP1 siRNA or negative control siRNA. Both the successful induction of SPP1⁺ TAM-like macrophages and siRNA knockdown efficiency were assessed by qPCR and western blotting, together with ELISA quantification (#SEA899Hu, USCN Life Science, China) of secreted SPP1/OPN in conditioned media. Macrophage conditioned medium (Mφ^CM^) was collected as described above, clarified by centrifugation, and used to treat tumor cells. Tumor cell migration was assessed using wound-healing and transwell invasion assays. For both assays, cells were cultured in serum-free RPMI-1640 medium supplemented with 25% Mφ^CM^ (v/v) from different groups.

### Tube formation assay

The Matrigel matrix (60 μL; BD Biosciences) was mixed with RPMI-1640 medium in a 1:1 ratio and added to each well of a 96-well plate on ice to prevent premature polymerization. The plate was incubated at 37 °C for gel formation. Subsequently, a human umbilical vein endothelial cell (HUVEC) suspension (50 μL; 6 × 10⁴ cells/well) was seeded onto the polymerized Matrigel and cultured in RPMI-1640 complete medium at 37 °C in a humidified atmosphere with 5% CO₂ for 4 h. After the incubation period, 50 μL of NF^CM^, CAF^CM^, CAF^CM^ + Imalumab, CAF^CM^ + IgG, or Mφ^CM^ from different groups was added to the respective wells. Images were acquired with a light microscope at 10× magnification. Quantitative analysis of total branching length, node count, junction number, and overall mesh area was performed using the “Angiogenesis” plugin in ImageJ, following an established protocol 30.

### Statistical analysis

All experiments were performed with at least three biological replicates. Data are presented as mean ± standard deviation (SD). Statistical analyses were performed using GraphPad Prism. Significant differences between two groups were determined using a two-tailed Student's t-test, while comparisons among multiple groups were assessed using one-way ANOVA. A *p*-value of less than 0.05 was considered statistically significant (*p < 0.05, **p < 0.01, ***p < 0.001, ****p < 0.0001).

## Results

### A single-cell expression atlas of laryngeal lesions

**Figure 1:**
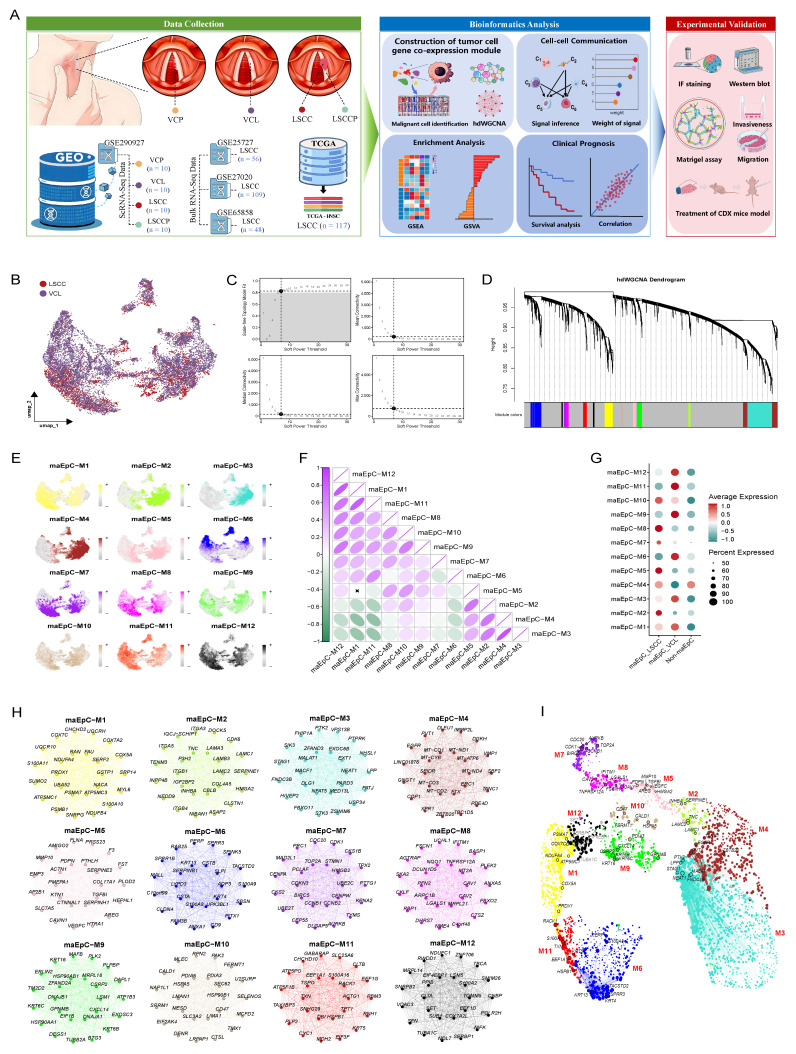
** Workflow and high-dimensional weighted gene co-expression network analysis (hdWGCNA) analysis for maEpCs. A** Schematic representation of the data used in this study, downstream bioinformatics analysis, and experimental flow. **B** Uniform Manifold Approximation and Projection (UMAP) plot visualizing maEpCs from vocal cord leukoplakia (VCL) and laryngeal squamous cell carcinoma (LSCC) samples, colored by sample origin. **C** Scale-free exponent and average connectivity for each soft threshold. **D** Dendrogram of gene clustering, with color bands indicating the assigned different modules. **E** UMAP projection of module eigengene showing the distribution characteristics of each module across maEpCs clusters. **F** Module correlogram showing the correlation strengths among identified gene modules. **G** Dot plot depicting the expression degree of modules in maEpCs from VCL and LSCC sources, and in non-maEpCs. **H** Co-expression network of the top 25 hub genes within each identified module. **I** Visualization of module-specific hub gene co-expression networks using UMAP.

Figure 1A outlines the overall workflow of this study. To comprehensively investigate tumor ecosystem heterogeneity during LSCC development and progression, we collected scRNA-seq data spanning normal laryngeal tissues to VCL and LSCC, along with bulk RNA-seq data from patients with LSCC, including comprehensive clinical information.

Following rigorous quality control and filtration of the scRNA-seq data, 302,558 single cells were retained for subsequent analysis. These included 69,576 cells from LSCCP, 57,620 from VCP, 103,447 from VCL, and 71,915 from LSCC (Fig. S1A, B). The Harmony algorithm was used to integrate cells from various tissue sources and remove batch effects. Based on canonical marker genes and manual annotation of the automated annotation results, eight known cell types were identified and subsequently projected into a two-dimensional space using Uniform Manifold Approximation and Projection (UMAP) (Fig. S1C). The bubble plot shows the specific expression of marker genes across defined cell types, validating the accuracy of cell type classification (Fig. S1D). Analysis revealed significant heterogeneity in the distribution, abundance, and proportions of these eight major cell types across tissues at different pathological stages (Fig. S1E).

Furthermore, for epithelial cells (EpCs), the initial copy number variation (CNV) levels across all regions were calculated using the InferCNV R package. Analysis of EpCs derived from the VCL and LSCC tissues revealed cells with abnormally elevated CNV levels, which were identified as EpCs (maEpCs) (Fig. S2A-D).

### Malignant epithelial cell co-expression network analysis

To explore the key gene regulatory architecture during LSCC occurrence and progression, we used hdWGCNA to analyze the co-expression network of maEpCs (Fig. 1B) and identified gene modules encapsulating coordinated transcriptional programs in the tumor. Network connectivity analysis revealed that a scale-free topology was optimally achieved at a soft-threshold power β of 7, and an unweighted cell network was constructed accordingly (Fig. 1C). The hdWGCNA dendrogram revealed multiple co-expressed gene modules identified using network evaluation, with 12 distinct gene modules (Fig. 1D). The UMAP plot illustrates the distribution characteristics of module-specific genes in tumor cells, revealing their differential expression characteristics in the TME (Fig. 1E). A heat map was used to visualize the correlations among the different modules, further elucidating the functional relationships between them (Fig. 1F). Module-trait association analysis revealed that the modules, maEpC-M2, M5, and M8, were significantly enriched in the maEpC_LSCC subgroup, indicating their potential involvement in tumor heterogeneity and progression (Fig. 1G). In contrast, the maEpC-M6, M9, M11, and M12 modules were predominantly enriched in the maEpC_VCL subgroup, indicating that leukoplakia development may be functionally associated with these modules. Notably, the maEpC-M1, M3, M7, and M10 modules were enriched in both maEpC subgroups, implying that these modules represent common characteristics of maEpC in VCL and LSCC (Fig. 1G). The top 25 hub genes from each module were extracted and visualized to evaluate their effects on tumorigenesis and cancer progression (Fig. 1H). In the maEpC_LSCC subgroup, modules maEpC-M2 and M5 were enriched with genes closely related to epithelial-mesenchymal transition (EMT) (*INHBA, HMGA2*, and *TNC*), extracellular matrix (ECM) remodeling (*MMP10, SERPINE1*, and *PDPN*), and hypoxia (*VEGFC, CAVIN1*, and *PLOD2*). However, hub genes in module maEpC-M8 were involved in EMT and hypoxia and participated in viral entry mechanisms (*CAV1/2, IFITM1*), which may be associated with the critical role of the human papillomavirus (HPV) in LSCC pathogenesis. In contrast, hub genes in the maEpC_VCL subgroup modules, maEpC-M6 and M9, were primarily involved in keratinocyte differentiation (*KRT16, SPRR3*, and *SPRR1B*), reflecting processes related to leukoplakia formation. The maEpC-M11 and maEpC-M12 modules were primarily associated with cellular energy metabolism, including oxidative phosphorylation and ATP synthesis (*NDUFC1* and *ATP5F1B*). Interestingly, among the shared modules, hub genes in maEpC-M7 included cell proliferation-related genes such as *CDK1, TOP2A*, and *CCNB1*, suggesting that maEpCs in both VCL and LSCC exhibit active proliferative capacity.

In the UMAP module, each gene was spatially arranged per its connection characteristics, showing the co-expression network of each module in two-dimensional space (Fig. 1I). The proliferation-related module, maEpC-M7, did not show any obvious connection with genes of any other module, suggesting that it may operate independently to participate in disease progression. The maEpC-M2, M5, and M8 modules, which were enriched in maEpC_LSCC cells, exhibited tightly interconnected internal relationships. The modules enriched in the maEpC_VCL subgroup were linked through maEpC-M1, with core genes such as PRDX1, COX5A, and NDUFA4 playing pivotal roles in oxidative phosphorylation, tumor cell apoptosis resistance, and proliferation. Herein, hdWGCNA analysis revealed modular characteristics of gene expression and association patterns across modules in LSCC and VCL-derived maEpCs. We observed that the development and progression of LSCC markedly alter the gene co-expression network within epithelial cells, characterized by a suppression of epithelial transcriptional states and the activation of pathways associated with tumor proliferation and mesenchymal phenotypes. This comprehensive systemic regulatory reprogramming provides a valuable theoretical framework for elucidating the molecular mechanisms driving LSCC progression. Furthermore, it offers critical insights for future functional annotation and mechanistic studies.

### POSTN^+^ fibroblasts are associated with LSCC progression

Using CellChat, we systematically mapped intercellular communication networks to uncover key interactions driving malignant progression. The analysis identified fibroblasts as the most prominent signaling source interacting with maEpCs in both VCL and LSCC (Fig. S3A, B).

**Figure 2:**
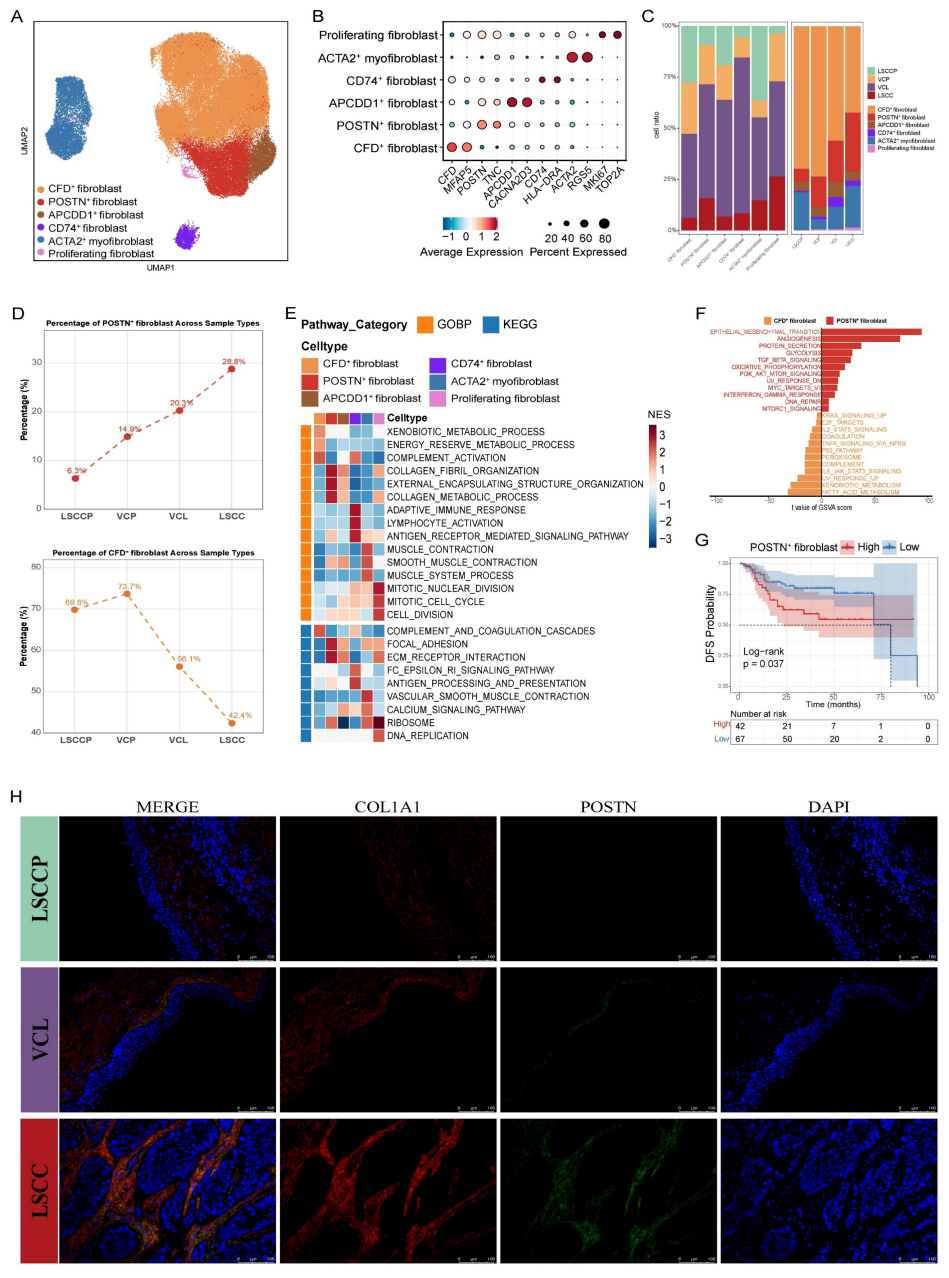
**Characterization of fibroblasts during LSCC progression. A** UMAP plot of fibroblast subsets colored by cell type. **B** Dot plots showing the expression of canonical marker genes across each fibroblast subset. **C** Bar plots showing the frequency (left) and proportion (right) of each fibroblast subset in different tissues. **D** Changes in infiltration proportion of POSTN^+^ fibroblasts (above) and CFD^+^ fibroblasts (below) in different tissues. **E** Heatmap displaying pathway activities of each fibroblast subset, quantified as normalized enrichment scores (NES) from Gene Set Enrichment Analysis (GSEA). **F** Pathway enrichment in POSTN^+^ versus CFD^+^ fibroblasts, assessed by GSVA score (two-sided unpaired limma-moderated t-test). **G** Kaplan-Meier curve showing disease-free survival (DFS) in the GSE27020 cohort, stratified by the optimal cut-off for POSTN^+^ fibroblast infiltration. **H** Representative immunofluorescence (IF) staining images of POSTN^+^ fibroblasts (COL1A1^+^ POSTN^+^ double positive) in LSCC, VCL, and non-malignant samples. Scale bar = 50 μm.

Fibroblasts, as key components of the tumor stroma, are actively involved in tumorigenesis and cancer progression. However, their heterogeneity in LSCC and its premalignant lesions remains elusive, hindering our understanding of the dynamic remodeling of the TME during LSCC development. To bridge this gap, we categorized fibroblasts into six subsets per their representative gene signatures (Fig. 2A, B; Table S6). Specifically, proliferating fibroblasts were defined by elevated expression of proliferation-associated genes such as *MKI67* and *TOP2A*; ACTA2^+^ myofibroblasts were marked by high expression of muscle-related contractile-related genes, including *ACTG2* and *RGS5*; CD74^+^ fibroblasts showed prominent expression of antigen-presenting genes such as *CD74* and *HLA-DRA*; APCDD1^+^ fibroblasts were characterized by expression of *APCDD1* and *CACNA2D3*; POSTN^+^ fibroblasts displayed high levels of ECM-related proteins, including POSTN and TNC; and CFD^+^ fibroblasts exhibited high expression of inflammatory genes (e.g., *CFD* and *MFAP5*). Among these subsets, CFD^+^ and POSTN^+^ fibroblasts exhibited opposite trends in abundance across LSCCP-VCL-LSCC groups (Fig. 2C, D). Particularly, the proportion of CFD^+^ fibroblasts decreased progressively, whereas that of POSTN^+^ fibroblasts increased consistently.

To further characterize the functionality of the different fibroblast subsets, we performed Gene Set Enrichment Analysis (GSEA) against gene sets from the Gene Ontology Biological Process (GOBP) and Kyoto Encyclopedia of Genes and Genomes (KEGG) (Fig. 2E). Pathways including collagen fibril organization, external encapsulating structure organization, collagen metabolic process, and ECM-receptor interaction were significantly upregulated in POSTN^+^ fibroblasts but downregulated in CFD^+^ fibroblasts, suggesting that POSTN^+^ fibroblasts may promote the malignant progression of LSCC through ECM remodeling. Conversely, the complement activation pathway was markedly upregulated in the CFD^+^ fibroblasts and downregulated in the POSTN^+^ fibroblasts. In CD74^+^ fibroblasts, immune-related pathways, including adaptive immune response, antigen receptor-mediated signaling pathway, and antigen processing and presentation, were significantly enriched. As expected, proliferative pathways such as mitotic nuclear division, mitotic cell cycle, cell division, and DNA replication were notably enriched in the proliferating fibroblast subsets. Furthermore, ACTA2^+^ myofibroblasts showed a notable enrichment in pathways involved in muscle contraction. This includes processes related to muscle contraction, smooth muscle contraction, and overall muscle system dynamics. We compared the hallmark pathway activities of POSTN^+^ and CFD^+^ fibroblasts using Gene Set Variation Analysis (GSVA). The results revealed a strong enrichment of pathways associated with tumor progression and metastasis, such as EMT, angiogenesis, glycolysis, and TGF-β signaling, in POSTN^+^ fibroblasts, whereas the enrichment of UV response and complement pathways was significantly increased in CFD^+^ fibroblasts (Fig. 2F). Correspondingly, survival analysis revealed that high POSTN^+^ fibroblast gene signatures in patients with LSCC correlated with inferior disease-free survival (DFS; *p* = 0.037) (Fig. 2G). These results collectively support the pro-tumorigenic and pro-metastatic roles of POSTN^+^ fibroblasts in LSCC. Furthermore, IF staining confirmed that the proportion of POSTN⁺ fibroblasts increased during progression from LSCCP to VCL and then to LSCC, reinforcing the findings from the *in silico* analysis (Fig. 2H).

**Figure 3:**
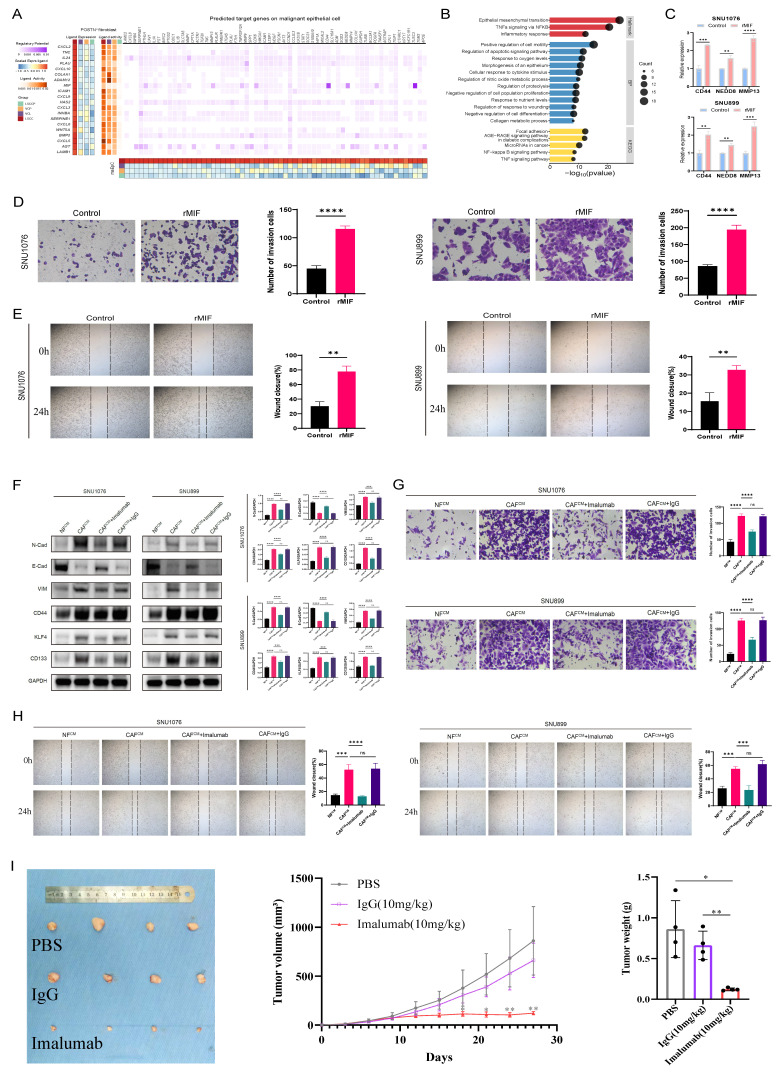
** Intercellular communication between POSTN^+^ fibroblasts and maEpCs in LSCC and validation of their functional. A** Heatmaps showing the regulatory patterns between POSTN^+^ fibroblasts and maEpCs based on NicheNet analysis. **B** Enriched Hallmark, Gene Ontology (GO), and Kyoto Encyclopedia of Genes and Genomes (KEGG) pathways among the NicheNet-predicted target genes in maEpCs. **C** qPCR analysis of target gene expression in SNU899 or SNU1076 cells with or without stimulation by rMIF (n = 3 per group). **D, G** Representative images of invaded SNU899 or SNU1076 cells from transwell invasion assays after 48 h of culture under the indicated experimental conditions. Images were acquired from randomly selected fields (n = 3 per group). **E**, **H** Representative images of wound-healing in SNU899 or SNU1076 cells cultured under the indicated experimental conditions at 0 and 24 h post-scratch (n = 3 per group). **F** SNU899 or SNU1076 cells were incubated for 48 h with different conditioned media (CM); epithelial-mesenchymal transition (EMT)-related and stemness-related protein expression was evaluated using immunoblotting (n = 3 per group). **I** Representative images of excised tumours (left panel), tumour volume quantification over time (middle panel), and tumour weight (right panel) from SNU1076 tumour-bearing mice following treatment with PBS, IgG, or imalumab (n = 4 per group). *p < 0.05; **p < 0.01; ***p < 0.001, **** *p* < 0.0001.

Calculation of interaction weights between fibroblast subsets and tumor cells across groups using the CellChat algorithm revealed that POSTN^+^ fibroblasts had the most frequent interactions with maEpCs in both VCL and LSCC (Fig. S3C, D). IF labeling revealed that in LSCC tissues, POSTN^+^ cells were in close proximity to tumor cells, providing spatial support for potential crosstalk between the two cell types (Fig. S3E). Therefore, to further investigate how these progressively increasing numbers of POSTN^+^ fibroblasts regulate tumor cells and the mechanisms via which they promote LSCC occurrence and development, we performed a multigroup NicheNet analysis. The results demonstrated that MIF was specifically upregulated in POSTN^+^ fibroblasts in the LSCC group and showed high ligand activity. The ligand MIF binds to receptors on tumor cells, thereby upregulating the expression of multiple tumor progression-related target genes, including *CD44, NEDD8*, and *MMP13* (Fig. 3A). Functional enrichment analysis of these target genes indicated significant enrichment in biological processes such as EMT, positive cell motility regulation, and collagen metabolic processes, as well as in the KEGG pathways related to focal adhesion and microRNAs in cancer (Fig. 3B). These findings further supported the pro-tumorigenic and malignancy-promoting regulatory roles of POSTN^+^ fibroblasts in tumor cells.

Next, to assess the pro-tumorigenic effects of MIF on LSCC, rMIF was used to directly stimulate LSCC cell lines (SNU899 and SNU1076). The results demonstrated that rMIF stimulation markedly upregulated the transcriptional levels of NicheNet-predicted target genes in tumor cells, including *CD44*, *NEDD8*, and *MMP13* (Fig. 3C). Concurrently, MIF treatment significantly enhanced the migratory and invasive capacities of both LSCC cell lines compared with the untreated control group (Fig. 3D, E). These findings support the gene regulatory events and pro-tumor phenotypes predicted by NicheNet analysis to be triggered by POSTN^+^ fibroblast-secreted MIF.

To further validate the functional significance of POSTN^+^ fibroblast-derived MIF in the TME, fibroblasts were isolated and cultured from both LSCC and adjacent normal tissues. SNU899 and SNU1076 cell lines were cocultured with CM derived from normal fibroblasts (NF^CM^) or cancer-associated fibroblasts (CAF^CM^). Western blot analysis revealed that compared to the NF^CM^ group, the CAF^CM^ group exhibited significantly upregulated expression of stem cell and EMT markers (such as N-cadherin and vimentin) in tumor cells, along with downregulated expression of the epithelial marker, E-cadherin (Fig. 3F). Notably, when imalumab was used to neutralize MIF, the CAF^CM^-induced EMT and stem-like phenotypes were markedly reversed (Fig. 3F). Functionally, wound-healing and invasion assays consistently demonstrated that compared to NF^CM^, CAF^CM^ significantly enhanced the migratory and invasive capacities of LSCC cells, an effect that was similarly suppressed by MIF blockade (Fig. 3G, H). Moreover, consistent with the *in vitro* observations, imalumab treatment significantly suppressed tumor growth *in vivo*, as evidenced by reduced tumor growth rates as well as smaller final tumor volumes and weights compared with the PBS or IgG groups (*p* < 0.01) (Fig. 3I). Collectively, these findings indicate that MIF secreted by fibroblasts (CAFs) within the TME promotes stemness and EMT programs in LSCC cells, thereby enhancing their migratory and invasive properties and driving tumor progression. Targeting the MIF signaling axis may therefore represent a promising therapeutic strategy for interrupting this malignant CAF-tumor cell crosstalk.

### Crosstalk between endothelial cells and POSTN^+^ fibroblasts promotes angiogenesis

**Figure 4:**
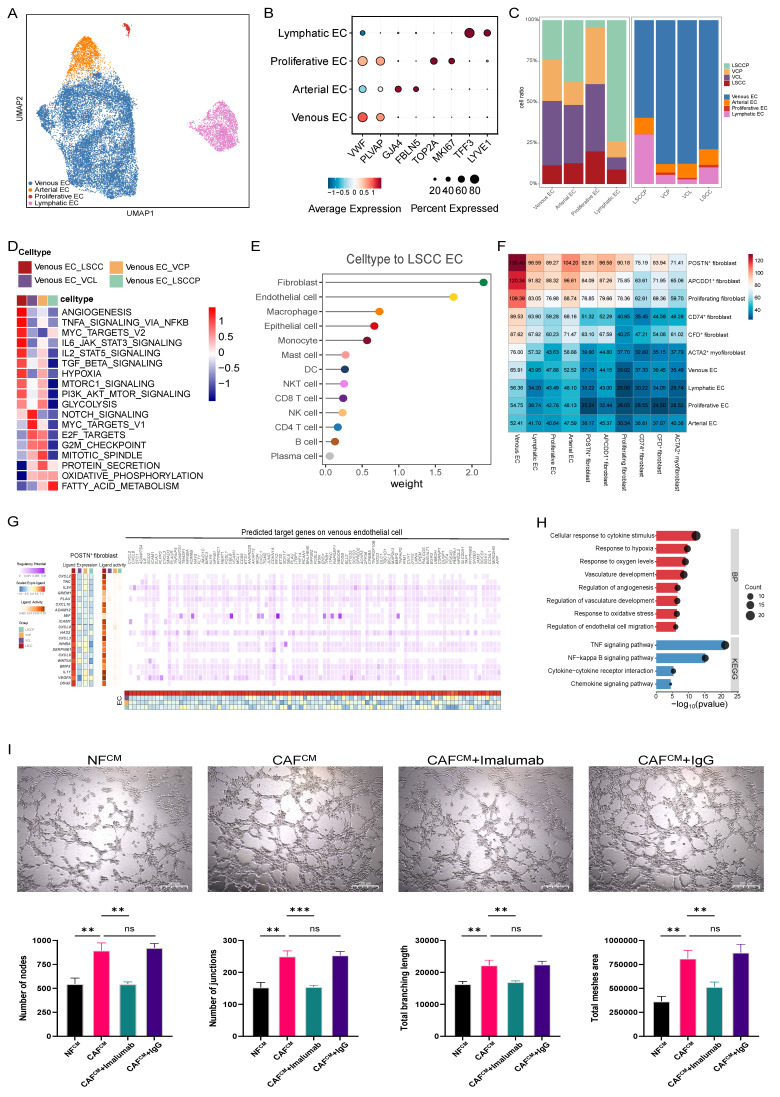
** Detailed characterization of endothelial cells (ECs) and angiogenesis features during LSCC progression. A** UMAP plot of ECs within each subset. **B** Dot plots showing the expression of canonical marker genes across each EC subset. **C** Bar plots showing the frequency (left) and proportion (right) of each EC subset in different tissues. **D** Heatmap displaying GSVA-based activity scores of Hallmark pathways across different EC subsets. **E** Comparison of interaction strength between different cell types and ECs in LSCC. **F** Heatmap representing the number of predicted ligand-receptor pairs between each fibroblast and EC subset in LSCC. **G** Heatmaps showing the regulatory patterns between POSTN^+^ fibroblasts and venous ECs based on NicheNet analysis. **H** Enriched GO and KEGG pathways among the NicheNet-predicted target genes in venous ECs. **I** Representative images showing the angiogenic capacity of HUVECs following 4 h culture in different CM (n = 3 per group). Scale bar = 200 μm. *p < 0.05; **p < 0.01; ***p < 0.001.

Abnormal angiogenesis occurs during laryngeal preneoplasia and persists throughout carcinogenesis 31. To further explore the angiogenic mechanism underlying LSCC occurrence and development, endothelial cells (ECs) were isolated and subjected to additional dimensionality reduction, clustering, and subgrouping. Among these subpopulations, we identified four EC subsets corresponding to traditional vascular beds: venous EC (*VWF*, *PLVAP*), arterial EC (*GJA4*, *FBLN5*), lymphatic EC (*TFF3*, *LYVE1*), and a proliferative EC subset characterized by the co-expression of venous and proliferative (*TOP2A*, *MKI67*) markers (Fig. 4A, B). Strikingly, proliferative ECs were observed in VCP, VCL, and LSCC tissues but not in LSCCP tissues, suggesting their potential relevance in tissue inflammation and tumorigenesis. (Fig. 4C).

To better understand the functional heterogeneity of venous ECs across different groups, we performed GSVA to compare their biological functions and signaling pathway activities using hallmark pathway sets. The results revealed that venous ECs in LSCC exhibited the most pronounced angiogenesis signature, whereas those in the VCL and VCP groups showed enrichment in proliferation-related pathways, such as MYC targets V1, E2F targets, G2M checkpoint, and the mitotic spindle pathways, indicating a highly proliferative phenotype. Angiogenesis is a cancer hallmark that plays a critical role in LSCC initiation and progression, a process that involves dynamic interactions between ECs and the surrounding TME 32. Therefore, we analyzed the communication network between ECs and various other cell types to gain insights into the mechanisms that promote angiogenesis. Similar to maEpCs, fibroblasts were identified as the most prominent signaling source interacting with ECs in both the VCL and LSCC (Fig. 4E, F; Fig. S3F-H). As expected, POSTN^+^ fibroblasts exhibited the highest interaction frequency with venous ECs in these tissues, which aligns with their biological role in pro-angiogenic functions (Fig. 2F). Subsequently, using multigroup NicheNet analyses, we further uncovered the potential mechanisms via which POSTN^+^ fibroblasts regulate venous ECs and promote angiogenesis. In the LSCC group, ligands derived from POSTN^+^ fibroblasts, including VEGFA, IL-24, and MIF, showed the highest activity. These ligands mediate signaling that upregulates angiogenesis-related target genes in venous ECs (Fig. 4G), thereby activating angiogenesis-associated pathways, including responses to hypoxia, vascular development, and angiogenesis regulation (Fig. 4H). Surprisingly, the tube formation assay demonstrated that compared with NF^CM^, CAF^CM^ significantly enhanced tube formation. This pro-angiogenic effect was reversed by imalumab, indicating that blocking of MIF signaling in CAFs can inhibit their pro-angiogenic potential (Fig. 4I). Based on these results, MIF, a ligand expressed by POSTN^+^ fibroblasts in LSCC, was found to mediate key interactions with both maEpCs and venous ECs, which are linked to tumor progression. Therefore, MIF may be a promising therapeutic target for LSCC development and progression.

### CD8^+^ T cell exhaustion occurs in LSCC and as early as the VCL stage

**Figure 5:**
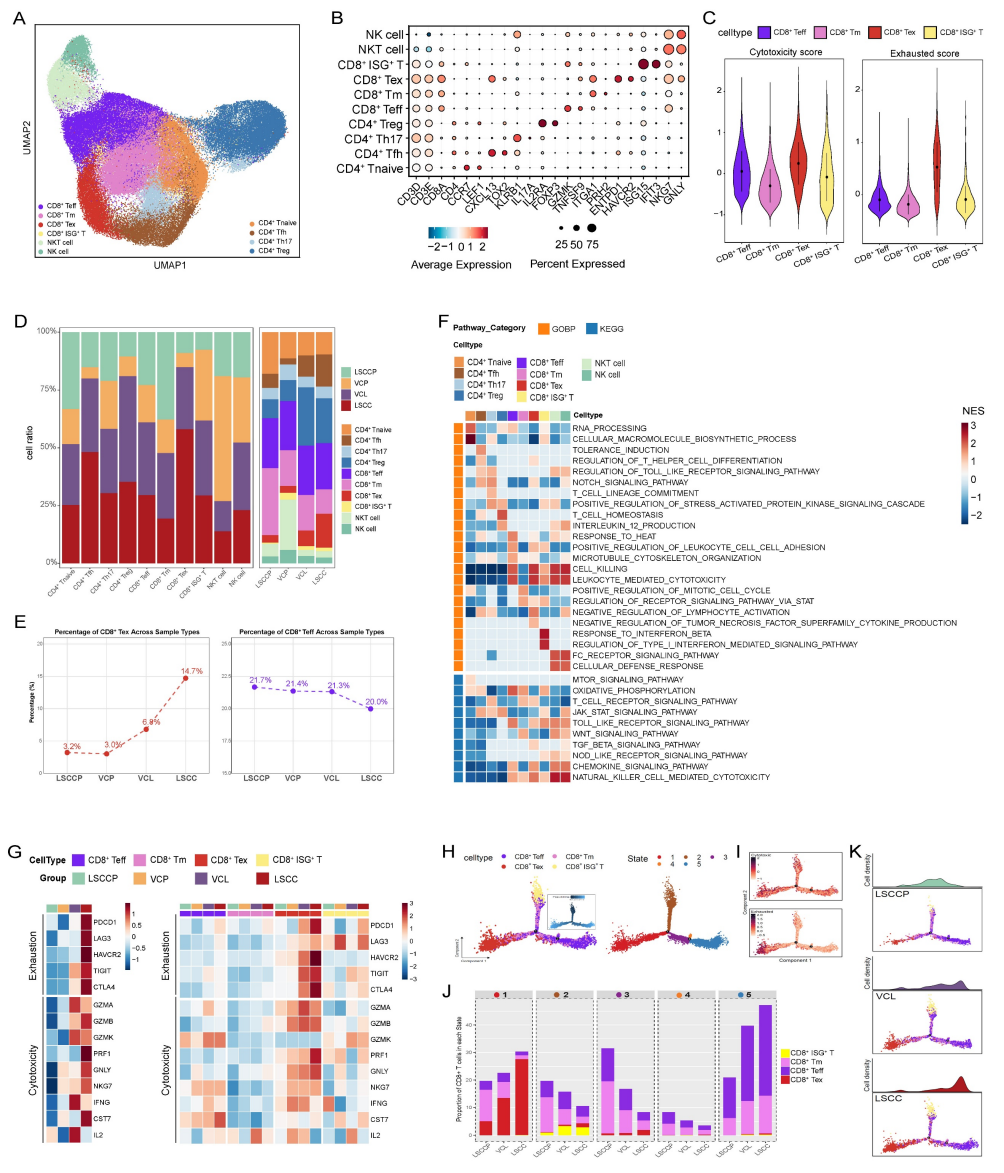
** Characterization of T cells during LSCC progression. A** UMAP plot of T cell subsets colored by cell type. **B** Dot plots showing the expression of canonical marker genes across each T cell subset. **C** Violin plots showing the cytotoxicity and exhausted scores for CD8^+^ T cell subsets, with colors representing different cell types. **D** Bar plots showing the frequency (left) and proportion (right) of each T cell subset in different tissues.** E** The infiltration proportion of CD8^+^ Tex (left) and CD8^+^ Teff (right) cells. **F** Heatmap displaying pathway activities of each T cell subset, quantified as normalized enrichment scores (NES) from GSEA. **G** Heatmap illustrating the expression of selected genes related to exhaustion and cytotoxicity across CD8^+^ T cell subsets. **H** Pseudotime trajectory analysis of CD8^+^ T cells, with subsets and distinct cellular states color-coded. The inset plot colors cells by pseudotime score, ranging from dark blue (early state) to light blue (terminal state). **I** Pseudotime plots demonstrating the temporal variation of cytotoxic (upper panel) and exhausted (lower panel) signatures within CD8^+^ T cells. **J** Stacked bar graph showing the distribution proportions of CD8^+^ T cells in each state across LSCC precursor (LSCCP), VCL, and LSCC groups. **K** Pseudotime plots of CD8^+^ T cells from LSCCP (top), VCL (middle), and LSCC (bottom) samples, with cell density along the pseudotime trajectory displayed along the upper section.

To explore alterations in the TIME throughout LSCC progression, we conducted further analysis on T cells and natural killer cells, which play central roles in anti-tumor responses. Based on marker gene expression, distinct subsets of CD8^+^ T cells were identified, including CD8^+^ effector T cells (CD8^+^ Teff; GZMK, TNFSF9), CD8^+^ memory T cells (CD8^+^ Tm; ITGA1, PRH2), CD8^+^ exhausted T cells (CD8^+^ Tex; ENTPD1, HAVCR2), and CD8^+^ ISG^+^ T cells (ISG15 and IFIT3) (Fig. 5A, B) 33. Using cytotoxicity and exhaustion scores calculated for each CD8^+^ T cell subset (Table S1), we observed that CD8^+^ Teff and CD8^+^ Tex cells exhibited high cytotoxicity scores, whereas CD8^+^ Tex cells showed the highest exhaustion level (Fig. 5C). These results confirmed the identity of each cellular subset. Furthermore, among these subsets, CD8^+^ Teff and CD8^+^ Tex cells exhibited opposing abundance trends during LSCC progression; the number of CD8^+^ Teff cells decreased progressively, whereas that of CD8^+^ Tex cells increased, and it showed significant infiltration as early as the VCL stage (Fig. 5D, E), suggesting that features consistent with an immunosuppressive state begin to emerge in the TME as early as the VCL stage, which was further exacerbated during progression to LSCC.

For CD4^+^ T cells, we identified CD4^+^ naïve T cells (CD4^+^ Tnaive; CCR7, LEF1), CD4^+^ follicular helper T cells (CD4^+^ Tfh; CXCL13, TOX2), CD4^+^ Th17 (KLRB1, IL17A), and Treg (IL2RA, FOXP3) (Fig. 5A, B) 33. GSEA revealed that CD4^+^ T naïve cells were significantly enriched in biological processes such as RNA processing and cellular macromolecule biosynthetic processes. CD4^+^ Tfh cells were enriched in immune tolerance-related pathways, including tolerance induction and regulation of T helper cell differentiation. Correspondingly, CD4^+^ Th17 and Treg cells were enriched in the T cell receptor signaling pathway and T cell homeostasis, respectively (Fig. 5F). Intriguingly, a substantial number of Tregs were detected in both VCL and LSCC tissues (Fig. 5E). Moreover, the proportion of CD4^+^ Tfh cells increased progressively with LSCC progression (Fig. 5E), further supporting the immunosuppressive state of the VCL and LSCC TME.

To better characterize the functional status of CD8^+^ T cells among various groups, we evaluated the expression levels of key exhaustion and cytotoxicity markers (Fig. 5G). The results showed that immune checkpoint genes, including *PDCD1, TIGIT, CTLA4, HAVCR2,* and* LAG3*, were elevated in CD8^+^ T cells from the LSCC group. Remarkably, significant upregulation of *TIGIT* and *CTLA4* was evident in CD8^+^ T cells from the premalignant VCL group, suggesting the early establishment of an immunosuppressive microenvironment. Further comparisons among CD8^+^ T cell subsets across groups revealed that genes encoding checkpoint molecules were highly expressed in CD8^+^ Tex cells from both the VCL and LSCC groups, with TIGIT showing particularly prominent expression. Interestingly, analysis of cytotoxicity markers revealed distinct functional patterns. While CD8^+^ T cells in LSCC showed high expression of cytotoxicity-related genes, such as *GNLY, NKG7, PRF1, CST7*, and *GZMB*, their counterparts in VCL predominantly expressed *IL2, IFNG, GZMK*, and *GZMA*.

Furthermore, we used Monocle to infer the state trajectories and investigate the dynamic transitions of CD8^+^ T cells during disease progression. This analysis revealed that CD8^+^ ISG⁺ T cells occupied the trajectory origin, while CD8^+^ Tex and most CD8^+^ Teff cells localized at terminal states (Fig. 5H). We observed that exhaustion signatures were upregulated during the transition to state 1. However, the cytotoxicity signatures, despite an initial increase, were downregulated throughout the trajectory (Fig. 5I). As anticipated, state 3, representing early intermediate CD8^+^ T cells, was mainly distributed in LSCCP samples, with limited presence at the terminal region of the cell state transition trajectory. Conversely, CD8^+^ T cells from VCL and LSCC were predominantly located in terminal states 1 and 5, corresponding to exhausted and cytotoxic effector states, respectively (Fig. 5J, K).

Our analysis of T cells confirms the immunosuppressive nature of the TIME in LSCC and provides the first evidence that such a strongly immunosuppressive state exists even at the precancerous stage (VCL), indicating that immune dysregulation is not just a result of cancer development but an early critical event that may facilitate malignant transformation. This finding challenges the traditional timeline of immune evasion in LSCC pathogenesis and underscores VCL as a crucial window for potential early immunopreventive interventions strategies.

### SPP1^+^ macrophages were identified as a pro-tumorigenic subset enriched in LSCC

**Figure 6:**
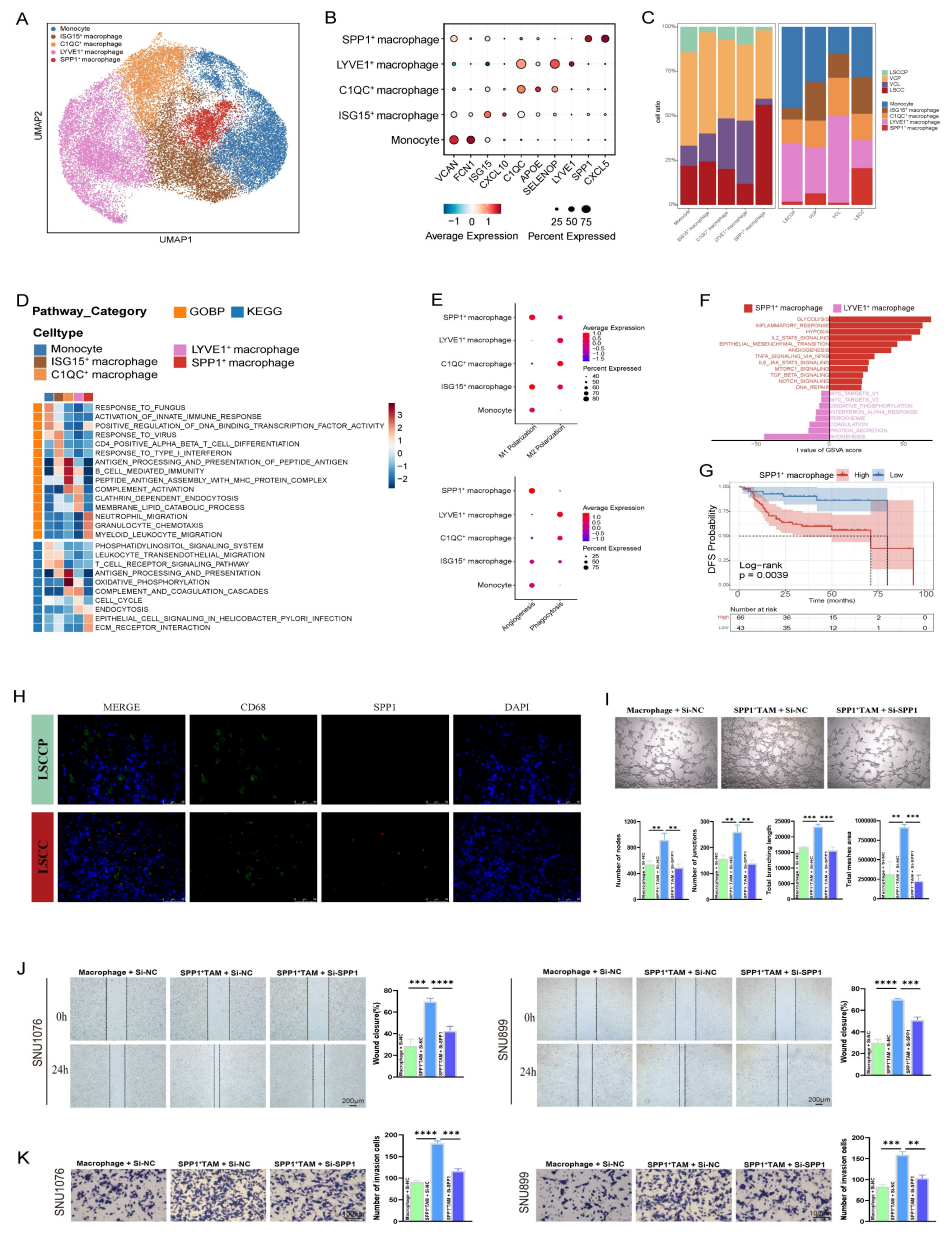
** Role of monocytes/macrophages in the immune functions during LSCC progression. A** UMAP plot of monocyte/macrophage cells colored by cell type. **B** Dot plots showing the expression of canonical marker genes across each monocyte/macrophage subset. **C** Bar plots showing the frequency (left) and proportion (right) of each monocyte/macrophage subset in different tissues. **D** Heat map displaying pathway activities of each monocyte/macrophage subset, quantified as NES from GSEA. **E** Dot plot depicting M1, M2, angiogenic, and phagocytic gene signatures across monocyte/macrophage subsets, shown as Z-score normalized log2(count + 1) values. **F** Pathway enrichment in LYVE1^+^ versus SPP1^+^ macrophages, assessed by GSVA score (two-sided unpaired limma-moderated t-test). **G** Kaplan-Meier curve showing DFS in the GSE27020 cohort, stratified by the optimal cut-off for SPP1^+^ macrophage infiltration. **H** Representative IF staining images of SPP1^+^ macrophages (CD68 and SPP1 double positive) within LSCC and LSCCP tissue sections. Scale bar = 50 μm. **I** Representative images showing the angiogenic capacity of HUVECs following 4 h culture in different CM (n = 3 per group). Scale bar = 200 μm. **J** Representative images of wound-healing in SNU899 or SNU1076 cells cultured with different CM at 0 and 24 h post-scratch (n = 3 per group).** K** Invasion of SNU899 or SNU1076 cells after 48 h of culture in different CM. Images showing representative randomly selected fields. (n = 3 per group). *p < 0.05; **p < 0.01; ***p < 0.001.

Myeloid cells were reclustered into monocytes/macrophages and dendritic cells (DCs) based on marker gene expression. To delineate the heterogeneity of monocytes/macrophages during LSCC progression, these cells were annotated into distinct subsets based on classical markers, including monocytes (VCAN and FCN1), ISG15^+^ macrophages, C1QC^+^ macrophages, LYVE1^+^ macrophages, and SPP1^+^ macrophages (Fig. 6A, B; Table S6). The proportions of these monocyte/macrophage subsets varied among samples derived from adjacent normal, precancerous, and cancerous tissues, with SPP1^+^ macrophages being significantly enriched in LSCC tissues (Fig. 6C). GSEA revealed distinct functional specializations among macrophage subsets; both C1QC^+^ and LYVE1^+^ macrophages exhibited significant activation of immune-related pathways. Particularly, C1QC^+^ macrophages were enriched in processes such as antigen processing and presentation and B cell-mediated immunity, whereas LYVE1^+^ macrophages were characterized by enhanced endocytosis. Complement activation was observed in both the C1QC^+^ and LYVE1^+^ macrophages. In contrast, SPP1^+^ macrophages demonstrated enrichment in tumor-promoting pathways, including ECM-receptor interaction (Fig. 6D).

Furthermore, the classical and alternative activation pathways of macrophages, commonly referred to as M1 and M2 polarization, respectively, highlight the phenotypic heterogeneity among different macrophage subsets. In line with previous reports, we observed that M1 and M2 polarization states were not mutually exclusive; both ISG15^+^ and SPP1^+^ macrophages co-expressed gene signatures associated with M1 and M2 phenotypes (Fig. 6E; Table S2) 29,34. Unexpectedly, immunocompetent C1QC^+^ and LYVE1^+^ macrophages exhibited a more pronounced M2 signature than M1 phenotype, suggesting that the phenotypic spectrum of TAMs in the TME of LSCC is more complex than that of the conventional M1/M2 dichotomy established *in vitro*. Moreover, C1QC^+^ macrophages, particularly LYVE1^+^ macrophages, exhibited markedly high phagocytic scores, whereas SPP1^+^ macrophages showed the highest angiogenic scores (Fig. 6E). These results aligned with the GSEA findings, which indicated anti-tumor attributes of LYVE1^+^ macrophages and pro-tumor characteristics of SPP1^+^ macrophages. To further explore the functional implications of this heterogeneity, GSVA was used to compare the activities of hallmark pathways in LYVE1^+^ and SPP1^+^ macrophages. This analysis revealed that pathways closely associated with tumor progression and metastasis, including glycolysis, hypoxia, EMT, and angiogenesis, were significantly activated in SPP1^+^ macrophages. In contrast, LYVE1^+^ macrophages were predominantly enriched in pathways related to protein secretion and myogenesis (Fig. 6F). Consistent with these pro-tumorigenic pathways, survival analysis showed that high expression of the SPP1^+^ macrophage gene signature significantly correlated with worse DFS in patients with LSCC (*p* = 0.0039) (Fig. 6G). Moreover, IF staining confirmed SPP1 expression in a subset of CD68-positive macrophages within LSCC tissues, which was nearly absent in LSCCP tissues (Fig. 6H). To validate the pro-tumorigenic function of SPP1⁺ macrophages, we next performed *ex vivo* functional assays. Briefly, SPP1⁺ TAM-like cells were induced by co-culturing M0 macrophages with LSCC cells, followed by siRNA-mediated SPP1 knockdown (si-SPP1) or transfection with a negative control (si-NC); and successful induction and knockdown efficiency were validated (Fig. S4A-C). The pronounced pro-angiogenic activity induced by Mφ^CM^ from si-NC SPP1⁺ TAM was largely abolished by Mφ^CM^ from si-SPP1 SPP1⁺ TAM, resulting in a marked reduction in HUVEC tube formation (Fig. 6I). Furthermore, wound healing and transwell assays demonstrated that, compared to the Mφ^CM^ from the si-NC group, the Mφ^CM^ derived from si-SPP1 SPP1⁺ TAM significantly attenuated the migratory and invasive capacities of LSCC cells (SNU1076 and SNU899) (Fig. 6J, K). Therefore, these direct functional experiments confirmed that SPP1⁺ macrophages form a pro-tumorigenic TAM subset within the LSCC TME and offer mechanistic support for the negative prognostic association.

### LAMP3^+^ DCs infiltrate from VCL to LSCC and exhibit a potent immunosuppressive phenotype

**Figure 7:**
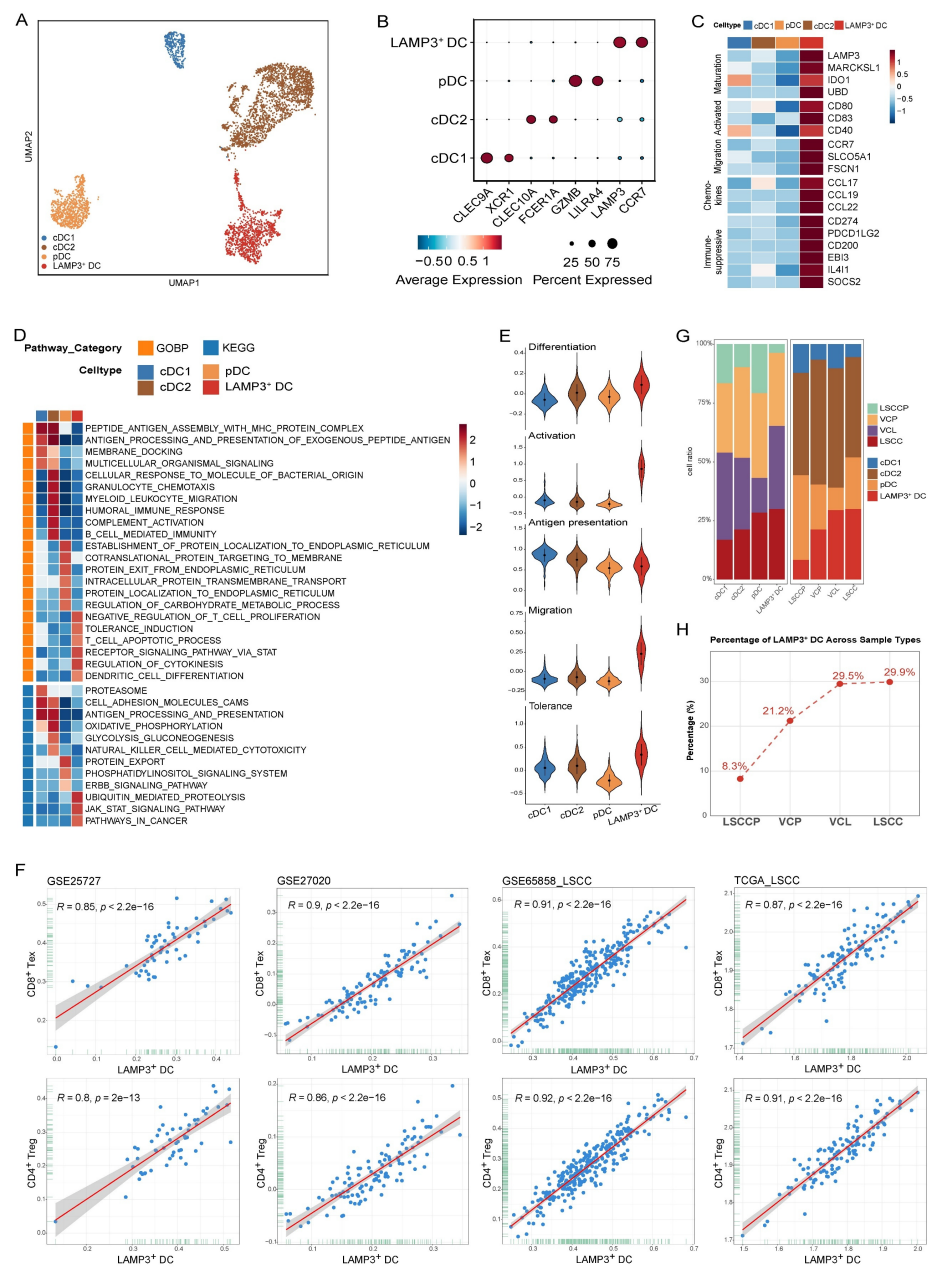
** Characterization of DCs during LSCC progression. A** UMAP plot of DCs colored by cell type. **B** Dot plots displaying characteristic gene expression within the various DC subsets. **C** Heatmap showing the normalized mean expression of genes associated with maturation, activation, migration, chemokines, and immunosuppressive scores in each DC subset. **D** Heatmap displaying pathway activities of each DC subset, quantified as NES from GSEA. **E** Violin plots showing the differentiation, activation, antigen presentation, migration, and tolerance scores of each DC subset. **F** Bar plots showing the frequency (left) and proportion (right) of each DC subset across various tissues. **G** Infiltration proportion of LAMP3^+^ DCs. **H** Correlation of CD8^+^ Tex or Treg signature with LAMP3^+^ DC based on bulk RNA-seq data. Each dot represents a patient (Pearson's correlations).

Based on marker gene expression, DCs were classified into distinct subsets, including conventional DCs (cDC1, cDC2, and LAMP3^+^ DC) and plasmacytoid DCs (pDC) (Fig. 7A, B; Table S6). Analysis of signature genes indicative of maturation (*LAMP3*, *MARCH1*, *IDO1*, and *UBD*), activation (*CD80*, *CD83*, and *CD40*), and migration (*CCR7*, *SLCO5A1*, and *FSCN1*) revealed that LAMP3^+^ DCs represent a DC subset with pronounced maturation, activation, and migratory features in LSCC (Fig. 7C and Table S3). Moreover, LAMP3^+^ DCs highly expressed specific chemokine ligands (CCL17, CCL19, and CCL22), which are known to recruit immune cells such as Tregs, helper T cells, and B cells via their corresponding receptors, CCR4 and CCRR7 (Fig. 7C)^35^,^36^.

GSEA analyses based on GOBP and KEGG gene sets revealed that antigen processing and presentation were significantly upregulated in cDC1 and cDC2 but downregulated in LAMP3^+^ DCs (Fig. 7D). Furthermore, signaling pathways, such as tolerance induction, T cell apoptotic process, and pathways in cancer were significantly enriched in LAMP3^+^ DCs compared to those observed in other DC subsets (Fig. 7D). Subsequent gene expression scoring in each DC subset demonstrated that LAMP3^+^ DCs displayed the highest differentiation, activation, and migratory scores, while exhibiting the lowest antigen presentation level (Fig. 7E). LAMP3^+^ DCs displayed the highest immune tolerance score (Fig. 7E), which was consistent with the upregulated expression of immunosuppressive genes, including *CD274 (PD-L1), PDCD1LG2 (PD-L2), CD200, EBI3, IL4I1*, and *SOCS2*, as well as the GSEA enrichment results (Fig. 7C, D). These results indicate that LAMP3^+^ DCs represent a subset of regulatory and tolerogenic DCs, which aligns with our previous observations in hypopharyngeal carcinoma and prior reports on other cancer types 29,37,38. This consistent phenotype suggests that LAMP3^+^ DC can suppress T cell activation and promote immune evasion within the LSCC TME.

To deepen understanding of the relationship between LAMP3^+^ DCs and T cell status, four bulk RNA sequencing datasets (GSE25727, GSE27020, GSE65858_LSCC, and TCGA_LSCC) were used for analysis. We computed signature scores representing LAMP3^+^ DC, Treg, and CD8^+^ Tex abundances and evaluated their correlations. Strikingly, we observed strong and consistent positive correlations between the LAMP3^+^ DC signature and both the Treg and CD8^+^ Tex signatures across all cohorts (Fig. 7F). Specifically, Pearson's R values ranged from 0.80 to 0.92, accompanied by statistically significant p-values (*p* < 0.0001). Taken together, these data imply that LAMP3^+^ DCs are intimately linked to both Tregs and exhausted CD8^+^ T cells within the TME. Based on their secretion characteristics and associated analyses, LAMP3+ DCs may play a significant role in the recruitment or local expansion of Tregs and contribute to T cell exhaustion. This process potentially fosters an immunosuppressive microenvironment.

We observed a progressive increase in the infiltration of LAMP3^+^ DCs from LSCCP to VCL and then to LSCC tissues. Importantly, LAMP3^+^ DC abundance reached considerably high levels as early as in VCL, which was nearly comparable to those observed in LSCC tissues (Fig. 7G, H). Considering these features of LAMP3^+^ DCs, we propose that LAMP3^+^ DCs may play a pivotal role in the early and ongoing formation of an immunosuppressive TME. The findings indicate that LAMP3^+^ DCs are crucial in tumor progression by promoting immune evasion from the early stages of carcinogenesis. They manipulate the TME, fostering immune suppression that enables tumor growth. This highlights the potential of targeting LAMP3^+^ DCs to modulate the TIME and enhance anti-tumor immune responses in cancer treatment.

## Discussion

The TME is a complex and dynamic ecosystem, the composition and cellular interactions of which profoundly influence tumor initiation and progression. Using scRNA-seq, the cellular heterogeneity and TME landscape of LSCC have been systematically characterized at unprecedented resolution. Key findings from these pioneering studies include the following: identification of spatially distinct tumor cell subpopulations within LSCC tumor nests—SPRR3-positive keratinized tumor cells located in the tumor core and MKI67-positive proliferative tumor cells situated at the tumor periphery; identification of abundant memory T and memory B cells that have not undergone immune activation in LSCC lymph node metastases; the observation that interactions between tumor cells and fibroblasts or myeloid cells are significantly more frequent than those with T or B lymphocytes; and the characterization of a cancer stem cell gene signature in LSCC, including markers such as *PROM1*, *ALDH1A1*, *SOX4*, *FOLR1*, and *DMBT1*. Zhou *et al.* 13 recently constructed the first single-cell atlas of VCP and VCL, providing a preliminary comparison of heterogeneity among tumor, T, B, and myeloid cells during LSCC progression from normal tissue to leukoplakia and primary carcinoma. They identified preexisting epithelial cell subpopulations with malignant potential in VCL. Furthermore, during the progression from VCL to LSCC, the functions of Tregs and regulatory B cells (Bregs) are reprogrammed to an immunosuppressive state, characterized by the upregulation of immunosuppressive genes, such as those belonging to the STAT family, *IL10*, and *TGFB1*, providing new insights into the mechanisms underlying the transition from VCL to LSCC. However, the dynamic evolution of the TME throughout the multistep progression from normal tissue through precancerous lesions to invasive carcinoma, particularly the conserved or specific oncogenic programs and aberrant intercellular signaling that commonly drive malignant transformation, has not yet been systematically elucidated.

**Figure 8:**
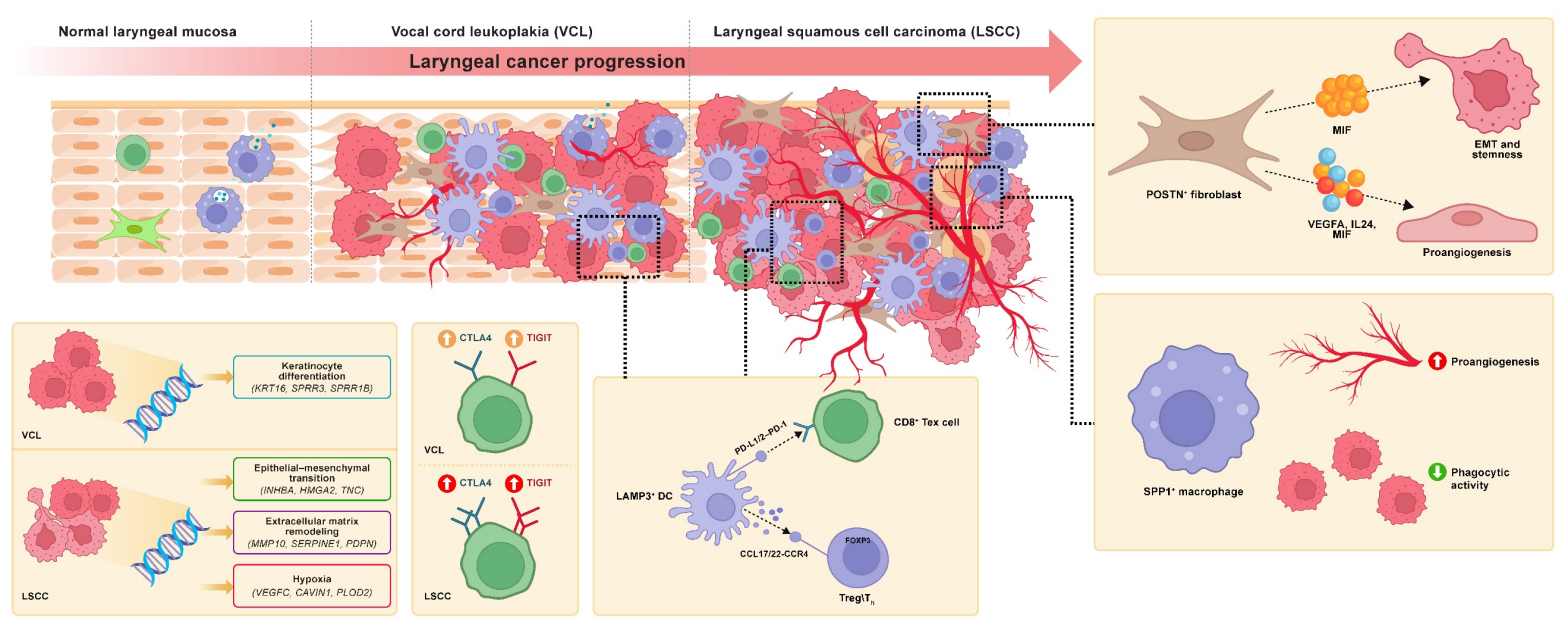
** Schematic illustration of tumor microenvironment remodeling characteristics during the progression from LSCCP to VCL and ultimately to LSCC**.

In this study, we systematically delineated the dynamic remodeling of the TME during LSCC progression at single-cell resolution using an in-depth analysis of a multi-stage scRNA-seq dataset comprising LSCCP, VCP, VCL, and LSCC samples, along with multiple LSCC bulk RNA-seq cohorts and functional experiment validation. We focused on tumor cells, fibroblasts, myeloid cells, and T cells, obtaining several significant findings for the first time within the LSCC ecosystem. First, using hdWGCNA, we revealed that LSCC-specific maEpC modules primarily drive EMT and hypoxia response processes during transcriptional reprogramming of maEpC development, whereas the proliferation module acts as an independent hub activated in both precancerous and cancerous stages, collectively driving the malignant transformation of EpCs. Moreover, we provided evidence regarding the pathogenic mechanism of HPV at the co-expression network level. Within the maEpC-specific modules, we observed that the core genes were significantly enriched in viral entry pathways, suggesting that HPV infection may drive the malignant progression of LSCC by remodeling the transcriptional network of epithelial cells. Second, we observed that POSTN^+^ and CFD^+^ fibroblasts exhibited opposite trends in infiltration proportions and biological functions during the transition from normal tissue to VCL and further to LSCC. Third, we identified SPP1^+^ macrophages as a pro-tumorigenic cell subset that was significantly enriched in LSCC tumor tissues, characterized by low phagocytic capacity and high angiogenic activity, and was associated with poor patient prognosis. Fourth, we revealed for the first time the central role of POSTN^+^ fibroblasts as key signaling hubs between cells. Via interactions with tumor cells via MIF ligands, they activate tumor stemness and EMT, promoting malignant progression. Additionally, POSTN^+^ fibroblasts interact with ECs through ligands, such as VEGFA and MIF, to induce angiogenesis in the TME. Fifth, we observed that CD8^+^ T cell exhaustion, characterized by upregulation of checkpoint molecules such as TIGIT and CTLA4, emerges as early as the precancerous VCL stage and is further exacerbated in LSCC, suggesting that an immunosuppressive microenvironment may already be established in the early stages of carcinogenesis. Sixth, we identified LAMP3^+^ DC as a potent immunosuppressive subset that persisted from the VCL to LSCC stages, showing significant positive correlations with Treg and CD8^+^ Tex cell abundance, suggesting that this population plays a crucial role in shaping the immune tolerance microenvironment during precancerous lesions and early stages of carcinogenesis. This study provides a detailed single-cell atlas of the multi-stage LSCC ecosystem, revealing the potential progression trajectories and cellular fate transition pathways within the microenvironment during LSCC evolution from normal tissue to precancerous lesions, and ultimately to malignant tumors (Fig. 8).

Our core findings included the progressively enhanced infiltration of POSTN^+^ fibroblasts during LSCC development and their multifaceted interactions with tumor cells and ECs via MIF-mediated signaling, which collectively remodeled the TME and promoted malignant progression. Within the tumor stroma, fibroblasts represent the predominant mesenchymal cell population and undergo dynamic evolution during tumor progression. Existing evidence indicates that CAFs exhibit high heterogeneity, comprise multiple subsets with distinct phenotypes and functions, and actively participate in tumor-promoting processes 39. Therefore, specific CAF subpopulations may act as potential targets for cancer therapy. However, the dynamic changes in the infiltration levels and functional phenotypes of different fibroblast subtypes during LSCC initiation and progression remain incompletely understood. This study revealed that POSTN^+^ fibroblast abundance increases progressively from normal laryngeal tissue to VCL and then to LSCC. A high POSTN gene signature in POSTN^+^ fibroblasts is associated with poor DFS in patients with LSCC, corroborating its tumor-promoting role. Periostin, encoded by POSTN, is predominantly expressed by CAFs and is minimally present in normal tissues. Previous studies have confirmed that POSTN promotes tumor cell adhesion and migration, contributes to cancer stem cell formation and the establishment of the pre-metastatic niche, and supports TME remodeling and tumorigenesis 40,41. Interestingly, He *et al.* 14 also observed a gradual increase in POSTN^+^ fibroblast infiltration during the progression of oral squamous cell carcinoma. In contrast, the proportion of CFD^+^ fibroblasts, characterized by inflammatory features and complement activation, progressively decreased with disease progression, suggesting that POSTN^+^ and CFD^+^ fibroblasts have distinct biological functions, as indicated by enrichment analysis. POSTN^+^ fibroblasts were significantly enriched in tumor-promoting pathways such as ECM organization, EMT, and angiogenesis, whereas CFD^+^ fibroblasts were more involved in immune regulatory processes such as complement activation.

The interactions between CAFs and tumor cells represent a core signaling component within the TME and have been observed across various cancer types as a pivotal mechanism promoting malignant progression 42. Using cell-cell communication analysis, we identified that POSTN^+^ fibroblasts are the most prominent signaling source interacting with tumor cells in both VCL and LSCC. This study is the first to provide evidence that potent interactions between POSTN^+^ fibroblasts and tumor cells begin at the VCL stage and become more pronounced in LSCC. Of note, multiNicheNet analysis revealed that MIF was the most critical ligand derived from POSTN^+^ fibroblasts in LSCC.

MIF is an enzyme-active inflammatory cytokine that promotes tumor cell proliferation by suppressing the tumor suppressor p53 43. In oral squamous cell carcinoma, MIF has been reported to facilitate the formation of myeloid-derived suppressor cells (MDSCs), thereby inhibiting the recruitment and anti-tumor functions of CD8⁺ T cells 44. Moreover, recent studies have revealed that MIF is overexpressed in various malignancies, including gastric cancer 45, melanoma 46, and prostate, bladder, and kidney cancer 47. Small-molecule inhibitors of MIF have been shown not only to directly suppress tumor cell growth but also to inhibit MDSC generation and reverse the TIME, thereby exerting dual anti-tumor effects 45,48. Bioinformatic analysis suggested that MIF derived from POSTN^+^ fibroblasts binds to receptors on tumor cells and upregulates tumor progression-related target genes, such as *CD44, NEDD8*, and *MMP13*. These genes were enriched in pathways associated with EMT, cell motility, and focal adhesion, suggesting that POSTN^+^ fibroblasts exert tumor-promoting and procarcinogenic effects via MIF signaling. Beyond this, POSTN^+^ fibroblasts also profoundly influence TME remodeling in LSCC through pro-angiogenic mechanisms. Abnormal angiogenesis is initiated at the precancerous stage of laryngeal lesions and persists throughout carcinogenesis 49. Consistently, we observed that communication between POSTN^+^ fibroblasts and venous ECs was the most active in both VCL and LSCC. Key ligands derived from POSTN^+^ fibroblasts in LSCC, such as VEGFA, IL-24, and notably MIF, bind to venous ECs and upregulate angiogenesis-related target genes, activating pathways, including hypoxia response and vasculature development. This prediction was further substantiated by functional validation. Direct stimulation of LSCC cell lines with rMIF activated the NicheNet-predicted target genes and elicited malignant phenotypes, whereas pharmacological neutralization of MIF with imalumab consistently reversed these effects across *in vitro* and *in vivo* models. This convergence of gain- and loss-of-function evidence establishes MIF as a core effector molecule linking CAF-derived signals to tumor-intrinsic aggressiveness and angiogenic remodeling in LSCC. Together, these findings for the first time reveal that fibroblast-derived MIF is a critical driver of malignant LSCC progression and highlight that targeting MIF signaling represents a promising therapeutic strategy capable of modulating both tumor-intrinsic programs and the TME.

Myeloid cells, especially macrophages, play a central role in regulating the TME and are involved in various aspects of tumor immunity and progression 50. In this study, monocytes/macrophages were classified into five distinct subpopulations. Among them, C1QC^+^ and LYVE1^+^ macrophages exhibited immunological activity, with significant activation of immune-related pathways, including antigen processing and presentation, endocytosis, and complement activation. LYVE1^+^ macrophages displayed a remarkably high phagocytosis score, suggesting their role as inflammatory macrophages with anti-tumor effects in LSCC. In contrast, SPP1^+^ macrophages were significantly enriched in the LSCC tissues. Although the tumor-promoting role of SPP1^+^ TAMs has been reported in various cancers, such as colon cancer 51, liver 52, and lung cancers 53. Their identity and function in LSCC remain poorly understood. Functional analysis revealed that SPP1^+^ macrophages exhibited a protumor phenotype, with significant enrichment in the ECM-receptor interaction pathway and elevated activity in classical tumor-related pathways, including glycolysis, hypoxia, EMT, and angiogenesis. Consistent with these findings, a high SPP1^+^ macrophage gene signature was significantly associated with poor prognosis in patients with LSCC. Importantly, we further validated the functional relevance of SPP1⁺ macrophages using *ex vivo* assays, providing direct experimental evidence that this macrophage subset actively drives malignant and pro-angiogenic behaviors in LSCC. Collectively, these findings establish SPP1^+^ macrophages as a pro-tumor macrophage subpopulation within the LSCC tumor microenvironment for the first time.

Furthermore, this study provides new evidence for the early establishment of an immunosuppressive microenvironment during LSCC pathogenesis. A notable finding was that CD8^+^ Tex significantly infiltrated precancerous VCL lesions, with this trend further intensifying in LSCC. Concurrently, the exhaustion phenotype, characterized by upregulation of checkpoint molecules such as TIGIT and CTLA4 in CD8^+^ T cells, can be observed as early as the precancerous VCL stage. Trajectory analysis revealed that CD8^+^ T cells derived from the VCL and LSCC were predominantly localized in the terminally exhausted state. Additionally, a substantial number of Tregs and a progressive decline in CD8^+^ Teff cells were observed during the transition from VCL to LSCC.

In parallel, a unique DC subset, LAMP3^+^ DC, was identified in LSCC that exhibited the lowest antigen-presenting capacity and the highest immune tolerance score. This DC subset resembles the LAMP3⁺ DCs first reported by Zhang *et al.* 38 in hepatocellular carcinoma, and our study is the first to confirm their presence and function in LSCC. Subsequent analyses across multiple independent bulk RNA-seq cohorts revealed strong positive correlations between LAMP3^+^ DC abundance and the Treg and CD8^+^ Tex signatures. More importantly, the infiltration level of LAMP3^+^ DCs in VCL tissues was already high and nearly comparable to that in LSCC, suggesting that LAMP3^+^ DCs promote the recruitment or local expansion of Tregs and participate in T cell exhaustion processes, thereby facilitating the formation of an immunosuppressive microenvironment at the precancerous stage. This discovery challenges the conventional timeline of immune evasion in LSCC, suggesting that the immunosuppressive features during the precancerous VCL stage may indicate a high-risk microenvironment for tumorigenesis, and underscores the importance of the VCL as a critical window for early immune intervention strategies.

Notably, the current diagnosis and risk stratification of VCL primarily rely on histopathological assessment of dysplasia, an approach that fails to accurately reflect the underlying molecular progression and immune microenvironment, limiting the precision of prognostic evaluation. Our study suggests that integrated assessment of key cellular features, including POSTN^+^ fibroblasts, SPP1^+^ macrophages, and LAMP3^+^ DCs, may provide a more comprehensive framework for assessing malignant transformation risk. From a translational perspective, future studies could integrate quantitative features derived from these cellular biomarkers—such as infiltration scores, gene expression signatures, or spatial distribution patterns—into multivariable statistical or machine learning-based models to generate a composite risk score for malignant transformation in VCL. Such an integrative approach may allow for better patient stratification, enhance early risk prediction, and ultimately guide personalized surveillance and treatment strategies for laryngeal precancerous lesions.

Although this study provides novel insights into the occurrence and development of LSCC, several limitations should be acknowledged. First, our analyses were based on a relatively comprehensive scRNA-seq dataset comprising 30 patients across different disease stages; however, the overall sample size remains limited, which may restrict the generalizability of the identified cellular biomarkers. Second, the cellular biomarkers identified in this study have not yet been validated in large patient cohorts. Future studies should therefore focus on validating these biomarkers in patient-derived samples to confirm their prognostic utility and robustness. Third, although our xenograft experiments demonstrated that pharmacological blockade of MIF using imalumab significantly suppressed tumor growth *in vivo*, additional studies employing immune-competent or carcinogen-induced LSCC models will be required to further elucidate the impact of MIF inhibition on tumor-immune microenvironment interactions and to better define its therapeutic potential in clinically relevant settings.

Building on existing research, this study systematically delineated a multi-stage single-cell atlas of cellular evolution and the molecular mechanisms underlying the development and progression of LSCC, comprehensively revealing the dynamic remodeling of the TME from normal tissue to precancerous lesions and subsequent progression to invasive carcinoma. Our findings underscore the pivotal role of POSTN^+^ fibroblasts in promoting LSCC malignancy and indicate that the interactive network formed among POSTN^+^ fibroblasts, tumor cells, and ECs via signaling ligands, such as MIF, serves as a core mechanism driving tumor progression and angiogenesis. Accordingly, MIF is a promising therapeutic target in LSCC pathogenesis. Additionally, this study revealed that T cell exhaustion and early infiltration of immunosuppressive DCs were already present during the precancerous stage, indicating the early formation of an immunosuppressive microenvironment. These findings improve our understanding of the LSCC ecosystem and its fundamental mechanisms, emphasize the significance of dynamic TME remodeling and early immunosuppressive microenvironment formation for future clinical evaluations and early intervention strategies, and provide potential strategic targets for both laryngeal precancerous lesions and LSCC, ultimately holding promise for improving clinical outcomes.

## Supplementary Material

Supplementary figures.

Supplementary tables.
