# Supplementary material for: Single-cell Deciphering of the Progression Trajectories of the Tumor Ecosystem in Laryngeal Squamous Cell Carcinoma
Source: Int J Biol Sci. 2026 Mar 28;22(7):3862–85. doi: 10.7150/ijbs.129291 (PMC13086088; doi:10.7150/ijbs.129291)
Supplement: Supplementary file 1 — Supplementary figures. [file ijbsv22p3862s1.pdf]

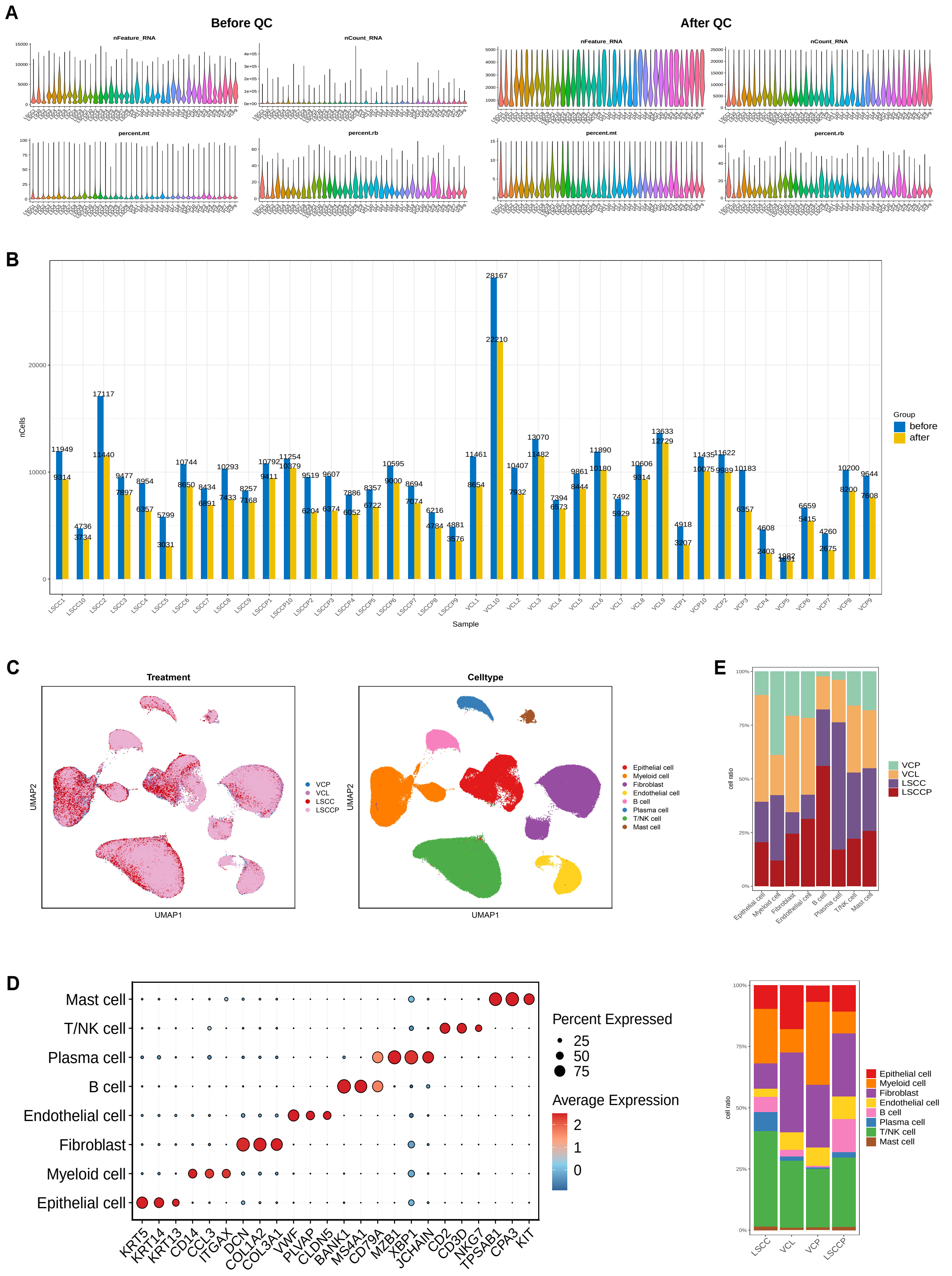

Figure S1. Quality control and subpopulation identification using single-cell sequencing data. A Violin plot showing the number of detected genes (nFeature), number of UMI (nCount), percentage of mitochondrial transcripts (percent.mt), and percentage of ribosomal gene transcripts (percent.rb) per single cell after quality control. **B** Bar chart showing the number of

cells in each sample before and after quality control. **C** UMAP plot of the clustering of 302,558 cells from all 10 LSCCP, 10 VCP, 10 VCL, and 10 LSCC tissue samples, color-coded by sample type (left) or cell type (right). **D** Dot plot representing marker gene expression across cell types. **E** Bar plots showing frequency (above) and proportion (below) of each cell type in different tissues.

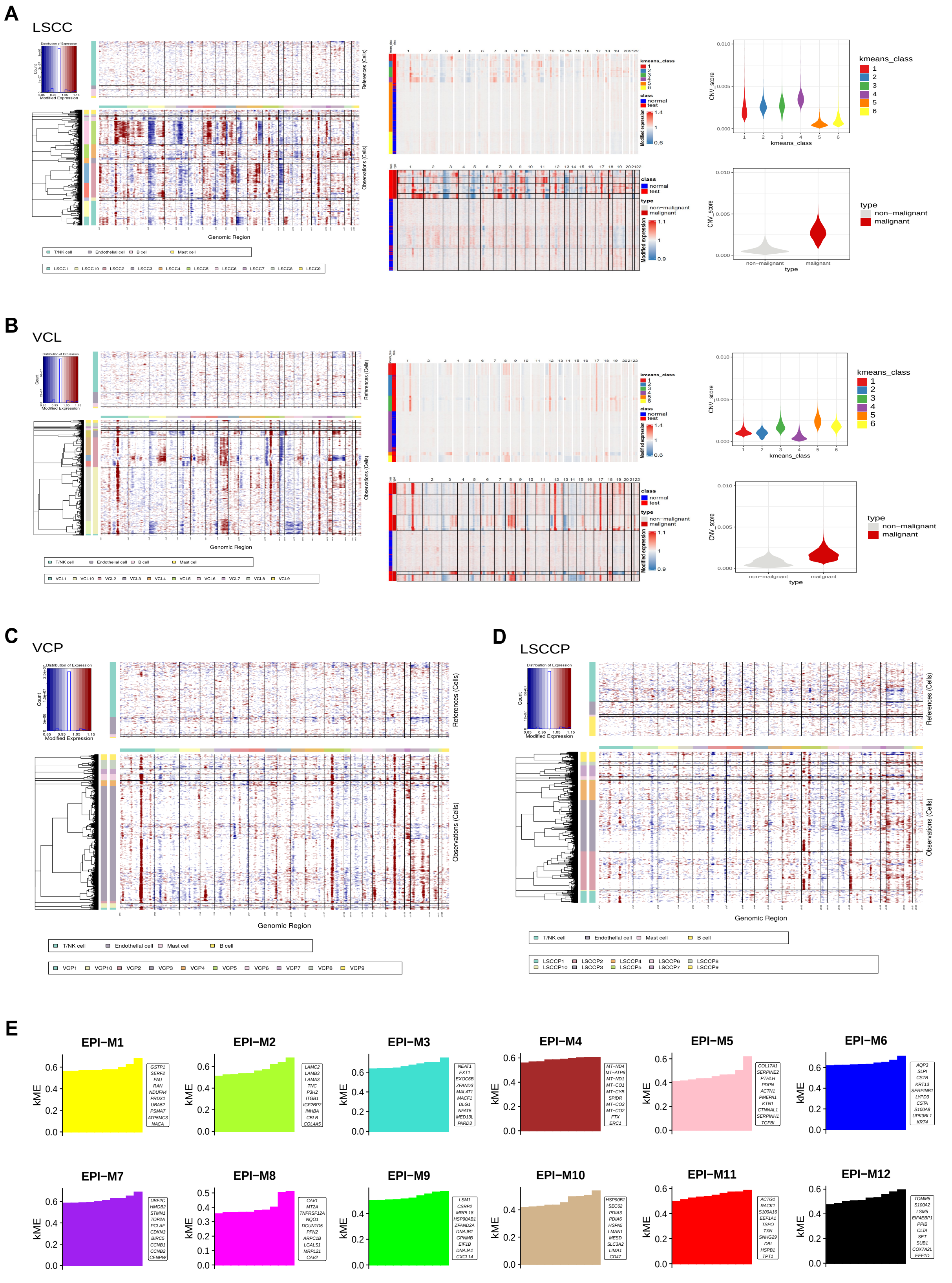

Figure S2. Identification and analysis of malignant epithelial cells. For the LSCC (**A**), VCL (**B**), VCP (**C**), and LSCCP (**D**) tissues, the InferCNV software was used to conduct copy number variation (CNV) analysis on the epithelial cells (left), K-means clustering analysis was conducted based on CNV scores for both epithelial cells and reference cells (middle), and CNV

scores were evaluated for each class derived from K-means clustering, as well as for the non-malignant or malignant epithelial cells identified (right). **E** Hubgenes of all modules identified by hdWGCNA, ranked by kME.

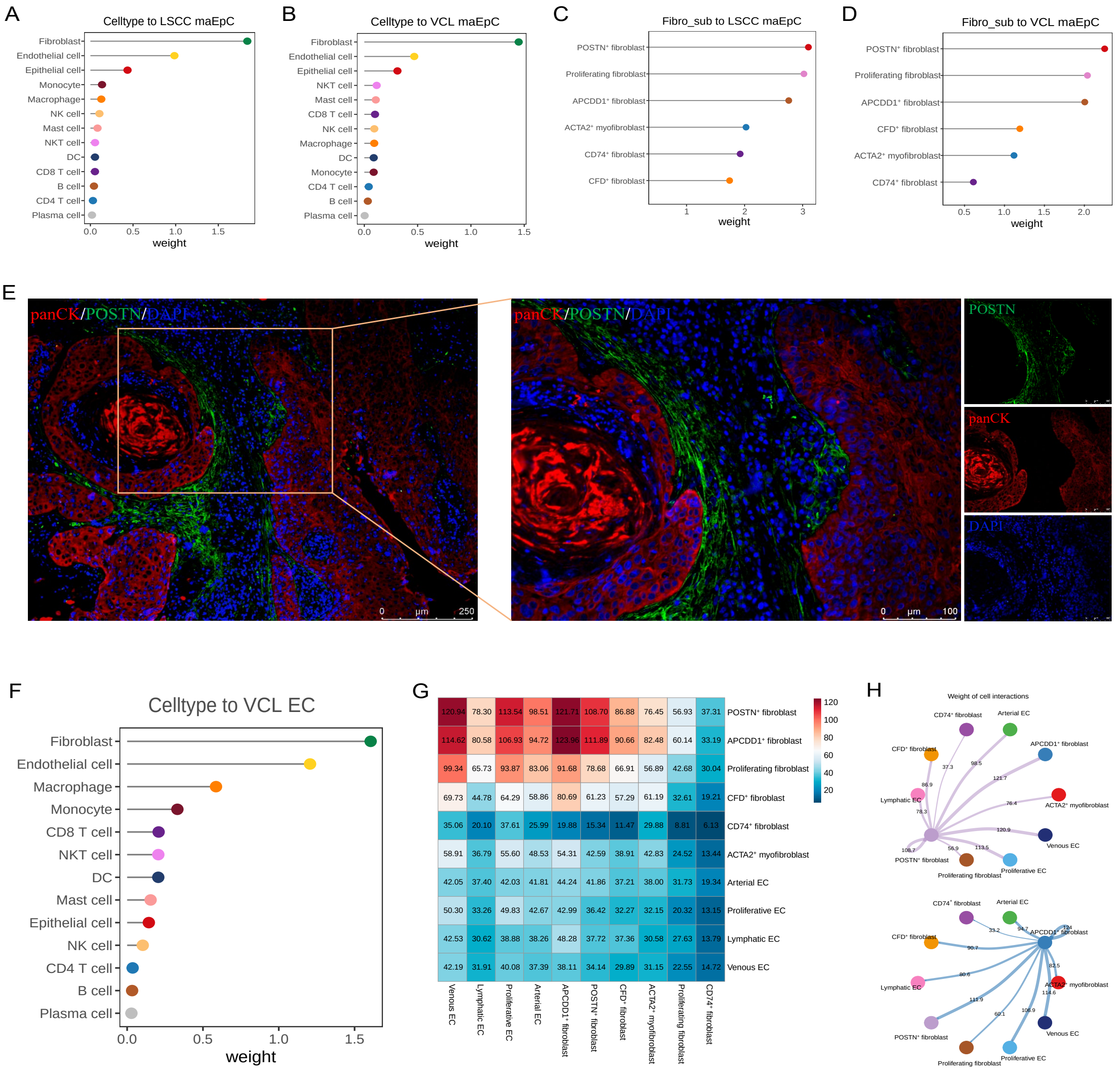

Figure S3. Intercellular communication between cells in LSCC or VCL. Comparison of interaction strength between different cell types and maEpC in LSCC (**A**) or VCL (**B**). Comparison of interaction strength between fibroblast subsets and maEpCs in LSCC (**C**) or VCL (**D**). **E** Representative IF staining images of POSTN and EPCAM molecules in LSCC tissue sections. **F** Comparison of interaction strength between different cell types and EC in VCL. **G** Heat map representing the number of predicted ligand-receptor pairs between each fibroblast and EC subset in VCL. **H** Chordal graphs show the weights of signals sent by POSTN^{+} fibroblasts or APCDD1^{+} fibroblasts to each EC subset in VCL.

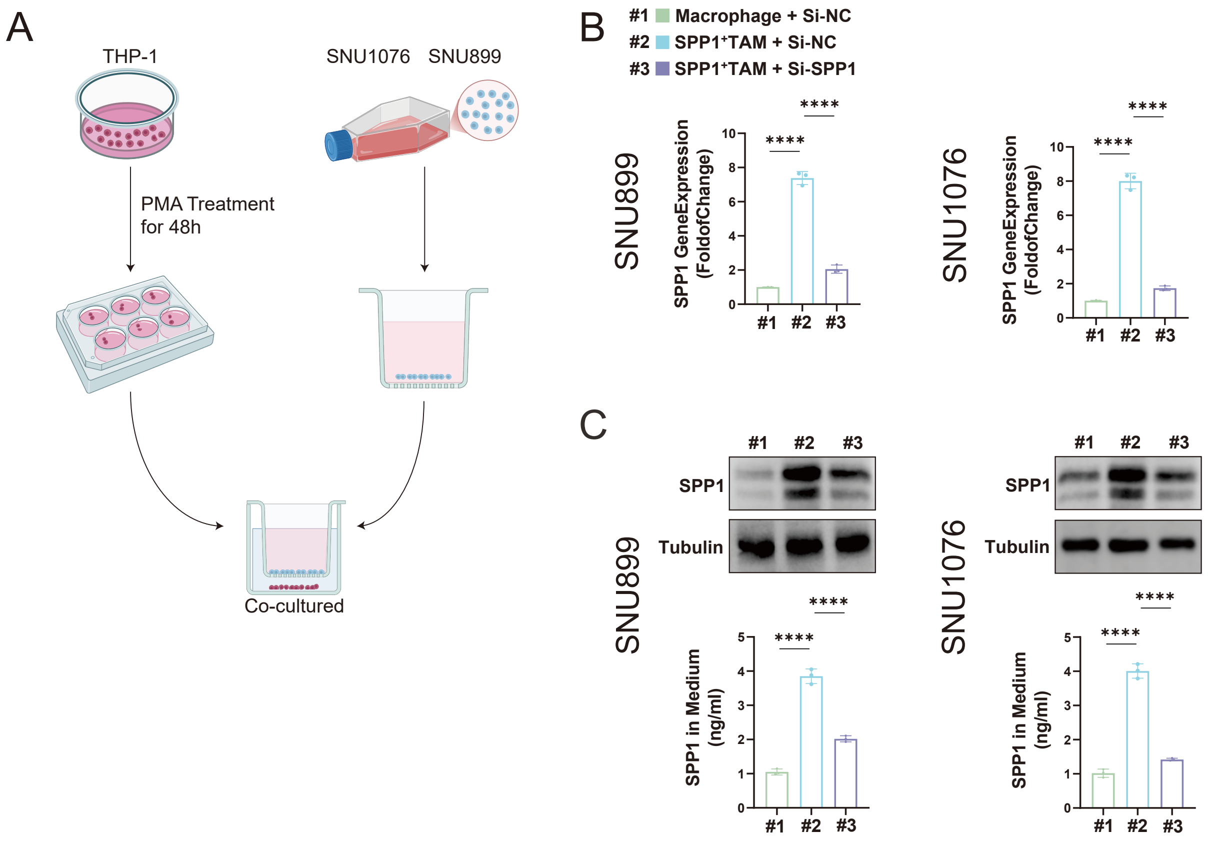

Figure S4. Generation and identification of SPP1⁺ tumor-associated macrophage (TAM)-like macrophages. A Schematic workflow of the generation and induction of SPP1⁺ TAM-like macrophages. **B** qPCR analyses of SPP1 expression in macrophages under the indicated conditions. **C** Western blotting (upper panel) was used to assess intracellular SPP1/OPN protein levels, while ELISA (lower panel) was performed to quantify SPP1/OPN secreted into the conditioned medium in each group.
